# *Clostridium difficile* Toxin A Undergoes Clathrin-Independent, PACSIN2-Dependent Endocytosis

**DOI:** 10.1371/journal.ppat.1006070

**Published:** 2016-12-12

**Authors:** Ramyavardhanee Chandrasekaran, Anne K. Kenworthy, D. Borden Lacy

**Affiliations:** 1 Department of Pathology, Microbiology and Immunology, Vanderbilt University School of Medicine, Nashville, TN, United States of America; 2 Department of Molecular Physiology and Biophysics, Vanderbilt University School of Medicine, Nashville, TN, United States of America; 3 Department of Cell and Developmental Biology, Vanderbilt University School of Medicine, Nashville, TN, United States of America; 4 Epithelial Biology Program, Vanderbilt University School of Medicine, Nashville, TN, United States of America; 5 The Veterans Affairs Tennessee Valley Healthcare System, Nashville, TN, United States of America; University of Illinois, UNITED STATES

## Abstract

*Clostridium difficile* infection affects a significant number of hospitalized patients in the United States. Two homologous exotoxins, TcdA and TcdB, are the major virulence factors in *C*. *difficile* pathogenesis. The toxins are glucosyltransferases that inactivate Rho family-GTPases to disrupt host cellular function and cause fluid secretion, inflammation, and cell death. Toxicity depends on receptor binding and subsequent endocytosis. TcdB has been shown to enter cells by clathrin-dependent endocytosis, but the mechanism of TcdA uptake is still unclear. Here, we utilize a combination of RNAi-based knockdown, pharmacological inhibition, and cell imaging approaches to investigate the endocytic mechanism(s) that contribute to TcdA uptake and subsequent cytopathic and cytotoxic effects. We show that TcdA uptake and cellular intoxication is dynamin-dependent but does not involve clathrin- or caveolae-mediated endocytosis. Confocal microscopy using fluorescently labeled TcdA shows significant colocalization of the toxin with PACSIN2-positive structures in cells during entry. Disruption of PACSIN2 function by RNAi-based knockdown approaches inhibits TcdA uptake and toxin-induced downstream effects in cells indicating that TcdA entry is PACSIN2-dependent. We conclude that TcdA and TcdB utilize distinct endocytic mechanisms to intoxicate host cells.

## Author Summary

*Clostridium difficile* is a bacterial pathogen that causes nearly half a million infections each year in the United States. It infects the human colon and causes diarrhea, colitis and, in some cases, death. *C*. *difficile* infection is mediated by the action of two large homologous toxins, TcdA and TcdB. Disruption of host cell function by these toxins requires entry into cells. There are multiple ways for pathogens and virulence factors such as viruses and toxins to enter host cells. The entry mechanism is often directed by a cell surface receptor and can impact the trafficking and virulence properties of the pathogenic factor. Investigating the internalization strategy can provide critical insight into the mechanism of action for specific pathogens and virulence factors. In our current study, we sought to determine the strategy utilized by TcdA to enter host cells. We show that TcdA uptake occurs by a clathrin- and caveolae-independent endocytic mechanism that is mediated by PACSIN2 and dynamin. We also show that TcdA and TcdB can utilize different routes of entry, which may have implications regarding their cytotoxic mechanisms. In summary, our results provide new insights into the mechanism of cellular intoxication by TcdA and the role of PACSIN2 in endocytosis.

## Introduction

*Clostridium difficile*, a gram-positive, spore-forming anaerobe, is the most common cause of healthcare-associated infections and gastroenteritis-associated death in the United States [1–3]. The pathogenesis of *C*. *difficile* is mediated by two large homologous exotoxins, TcdA and TcdB (308 kDa and 270 kDa, respectively), capable of causing epithelial cell death, fluid secretion and inflammation [4]. Recent studies, using isogenic single and double toxin knockout strains, have shown that either TcdA or TcdB alone can cause disease in animal models, with TcdB linked to severe disease phenotypes [5–7]. Most pathogenic isolates produce TcdA and TcdB emphasizing the need to consider both toxins when developing *C*. *difficile* therapeutics [8, 9].

TcdA and TcdB are broadly classified as AB toxins, wherein a B subunit is involved in the delivery of an enzymatic A subunit into the cytosol of a target cell. For *C*. *difficile* toxins, the A subunit is an N-terminal glucosyltransferase domain (GTD) that inactivates small GTPases, such as RhoA, Rac1 and Cdc42 [10, 11]. The B subunit is composed of the combined repetitive oligopeptides (CROPs) domain, delivery/pore-forming and autoprotease domains. The CROPs has been proposed to function as the receptor-binding domain because it can bind cell surface carbohydrates [12–14], and antibodies against the CROPs region of TcdA and TcdB can neutralize toxicity [15–17]. However, recent studies reveal that toxins lacking the CROPs domain can still bind, enter and perturb host cellular function, highlighting the presence of alternative or additional receptor binding regions within the toxins [18–21]. Upon binding to cells, toxins are taken up by endocytosis and transported to acidified endosomal compartments [4]. Acidification is thought to trigger a conformational change in the delivery domain, allowing it to insert into the membrane of the endosome and form a pore through which the enzymatic domains can be translocated [18, 22, 23]. Once inside the cytosol, host inositol hexakisphosphate binds the autoprotease domain to induce cleavage and release of the GTD [24]. The GTD transfers a glucose from UDP-glucose onto the switch I region of Rho family GTPases. This inactivation results in perturbation of the actin cytoskeleton and cell rounding (cytopathic effect) as well as apoptotic cell death (cytotoxic effect) [25–28]. At higher concentrations, TcdB is also capable of inducing aberrant production of reactive oxygen species, resulting in cell death by necrosis [29, 30].

Despite their homology, TcdA and TcdB appear to engage different receptors on the cell surface. Multiple receptors have been proposed for TcdA, including Galα1-3Galβ1-4GlcNac, rabbit sucrase-isomaltase and glycoprotein 96 [31–33]. Three recent studies have shown that poliovirus receptor-like protein 3, chondroitin sulfate proteoglycan 4, and frizzled proteins can function as TcdB receptors on epithelial cells [19, 34, 35]. Receptor binding by TcdB is followed by internalization via clathrin-dependent endocytosis [36], but the mechanism(s) by which TcdA enters cells has been less clear [36, 37].

In this study, we investigated TcdA cellular uptake by systematically perturbing the function of key host factors involved in various endocytic pathways using RNAi-based knockdown approaches and small molecule inhibitors, and by analyzing the toxin colocalization with markers of endocytic pathways by confocal microscopy. Our results indicate that cellular uptake of TcdA is mediated by a PACSIN2- and dynamin-dependent pathway and does not involve clathrin- or caveolae-mediated endocytosis.

## Results

### TcdA and TcdB utilize distinct endocytic mechanisms to intoxicate epithelial cells

**Fig 1:**
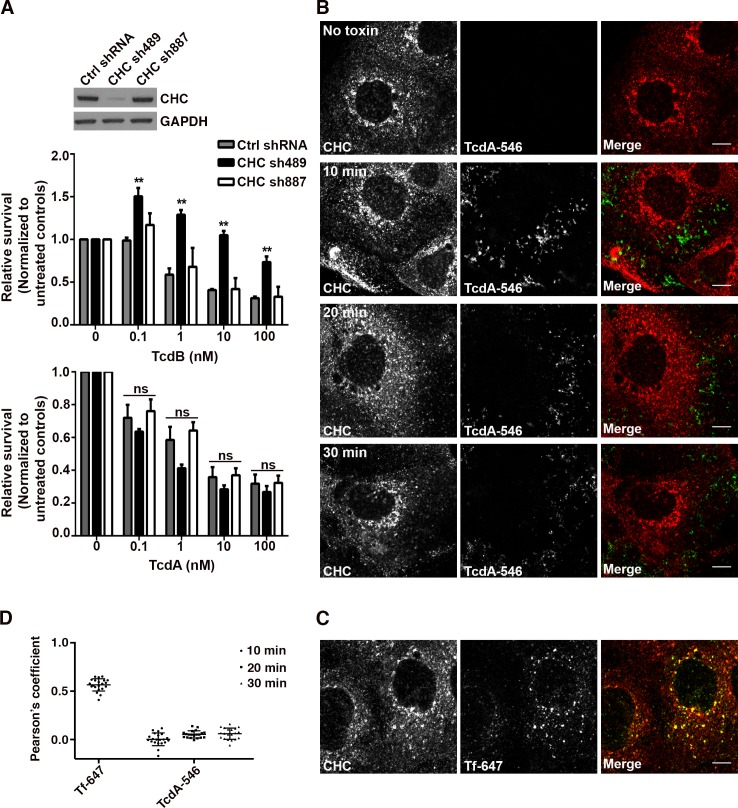
TcdA and TcdB utilize distinct endocytic mechanisms to intoxicate colonic epithelial cells. **(A) Depletion of clathrin heavy chain (CHC) does not affect TcdA-induced cytotoxicity.** Caco-2 cells expressing non-targeting shRNA (Ctrl shRNA) and shRNAs 489 and 887 targeting two different sequences in CHC were treated with indicated concentrations of TcdB or TcdA in triplicate. ATP levels were determined using CellTiterGlo and normalized to signal from untreated cells to assess the relative survival of cells post-toxin treatment. Results represent the mean and SEM of three independent experiments. Data were analyzed using two-way ANOVA and p-values were generated using Dunnett’s multiple comparisons test in GraphPad Prism. **p<0.005; ns, not significant. Western blot of whole cell lysates from shRNA-expressing Caco-2 cells were probed with antibodies against CHC and GAPDH (loading control). Expression of shRNA489 targeting CHC results in significant reduction in CHC protein levels as shown in the inset. **(B) TcdA does not colocalize with clathrin heavy chain during cell entry.** Caco-2 cells on glass coverslips were chilled at 10°C for 45 min and then exposed to media containing 50 nM TcdA-546 or buffer (no toxin control). The toxin was allowed to bind to cells for 45 min at 10°C. Unbound toxin was removed, and cells were shifted to 37°C to allow internalization of toxin for the times shown. At each time point, cells were washed once with pre-warmed PBS, fixed and stained for CHC, and imaged by confocal microscopy. Merged images show clathrin in red and toxin in green. Scale bars, 10 μm. (**C) Transferrin colocalizes with clathrin-coated pits as expected.** Experiment was performed as in (B) with 20 μg/ml of transferrin-alexa647 (Tf-647; positive control for colocalization with clathrin). Transferrin internalization occurred at 37°C for 10 min. Merged images show clathrin in red, transferrin in green and colocalization in yellow. Scale bars, 10 μm. The images shown in (B) and (C) are representative of multiple fields imaged from two independent experiments. **(D)** Pearson’s correlation coefficient to assess the extent of colocalization between clathrin heavy chain and Tf-647 or TcdA-546. Data represent mean and SD of 20 individual cells.

We first examined whether TcdA-induced cytotoxicity in colonic epithelial cells requires clathrin-mediated endocytosis (CME). Human colorectal adenocarcinoma (Caco-2) cells were transduced with non-targeting shRNA (ctrl shRNA) and shRNAs (sh489 and sh887) targeting two different sequences in the clathrin heavy chain (CHC). Expression of sh489 resulted in greater than 90% reduction in CHC protein levels, whereas sh887 did not alter CHC levels in cells (Fig 1A; inset). We challenged these shRNA-expressing cells with TcdA and TcdB concentrations ranging from 100 pM to 100 nM and assayed for cellular viability using CellTiterGlo. As expected, cells expressing sh489 (that were depleted of CHC) showed increased survival relative to cells expressing ctrl shRNA or sh887 when challenged with TcdB (Fig 1A; top panel). However, depletion of CHC did not affect TcdA-induced cell death across the range of concentrations tested (Fig 1A; bottom panel). A similar observation was made using a transient knockdown of CHC with small interfering RNA (siRNA) (S1 Fig). Taken together, these data show that clathrin heavy chain is dispensable for TcdA-induced toxicity in Caco-2 epithelial cells.

Our cytotoxicity data suggest that CME may not be required or involved in TcdA entry. To test this, we checked for colocalization of fluorescently labeled TcdA (TcdA-546) with labeled TcdB (TcdB-647) and CHC, markers of clathrin-mediated endocytosis. We verified that fluorescent labeling with Alexa dyes did not affect TcdA function prior to using TcdA-546 in our imaging assays (S2 Fig). For the confocal assays, we intoxicated cells with 50 nM of TcdA-546 as it provided sufficient signal and dynamic range needed for image analyses (S3 Fig). Confocal microscopy revealed minimal to no detectable colocalization of TcdA-546 with TcdB-647 during cell entry (S4 Fig). However, our labeling efficiency and signal intensity for TcdB was poor and less than desirable for colocalization and other imaging-based analyses. The technical challenges associated with obtaining higher labeling efficiencies while maintaining toxin function and internalization prevented us from using TcdB as a control in future immunofluorescence assays. As a result, in our subsequent experiment, transferrin (Tf-647) was used as a positive control for colocalization with CHC. As expected, Tf-647 exhibited significant colocalization with clathrin-positive vesicles (Fig 1C and 1D). However, in similar experiments, TcdA-546 did not colocalize with clathrin-positive structures during cell entry (Fig 1B and 1D). Taken together, our findings support a clathrin-independent mechanism of entry for TcdA and indicate that TcdA and TcdB utilize distinct endocytic mechanisms to intoxicate epithelial cells.

### Clathrin-independent uptake of TcdA requires functional dynamin

**Fig 2:**
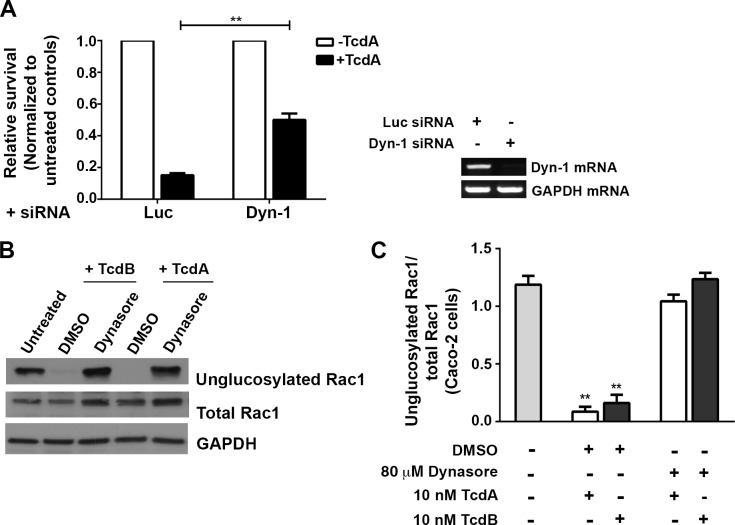
TcdA-induced Rac1 glucosylation and cytotoxicity are dynamin-dependent. **(A) Depletion of dynamin-1 confers resistance to TcdA challenge.** Caco-2 cells were transfected with 10 nM siRNA against dynamin-1 (Dyn-1) or luciferase (Luc; non-targeting control) and then intoxicated with 50 nM TcdA. ATP levels were determined using CellTiterGlo and normalized to signal from untreated cells to assess the relative survival of cells post-toxin treatment. The data represent the average of four independent experiments performed in triplicate with the SEM indicated as error bars. Data were analyzed using Welch’s t test. **p<0.005. RT-PCR confirms that siRNA treatment resulted in a decrease in Dyn-1 mRNA expression. **(B) Pharmacological inhibition of dynamin GTPases prevents Rac1 modification by TcdA**. Caco-2 monolayers were pretreated with either 80 μM dynasore or with an equal amount of DMSO control for 1 h at 37°C. Cells were switched to 4°C for 1 h and then intoxicated with either 10 nM TcdA or TcdB (positive control). Toxins were allowed to bind at 4°C for 1 h and then internalize at 37°C. TcdA and TcdB treated cells were harvested after 25 min and 15 min, respectively. Whole cell lysates were prepared for SDS PAGE and Western blot. The blot was probed with antibodies against the unglucosylated and total Rac1, and GAPDH. Cells that did not receive toxin or treatment were used as a control. **(C)** Four replicates of the experiments shown in (B) were quantified by densitometry and represented as the ratio of unglucosylated and total Rac1 levels. Results reflect the mean and SEM, and were analyzed using one-way ANOVA. p-values were generated using Dunnett’s multiple comparisons test in GraphPad Prism. **p<0.005.

Clathrin-independent endocytic (CIE) pathways can be dynamin-dependent or -independent [38, 39]. Dynamin is a large GTPase that facilitates scission and release of newly formed endocytic vesicles from the plasma membrane. To determine if the clathrin-independent uptake of TcdA requires dynamin function, we perturbed dynamin activity in cells by siRNA depletion or pharmacological inhibition and studied the effect on toxin-induced Rac1 glucosylation and cell death. Caco-2 cells were transfected with siRNAs targeting dynamin-1 or luciferase (non-targeting control) and subsequently challenged with TcdA. We found that depletion of dynamin-1 improved survival of cells treated with TcdA by at least three-fold compared to the luciferase control (Fig 2A). We verified that dynamin is important for the TcdA cytotoxic mechanism by using dynasore, a potent inhibitor of dynamin GTPases [40]. Dynasore treatment prevented Rac1 glucosylation by TcdB in Caco-2 cells, consistent with the known role of dynamin in CME (Fig 2B and 2C). Furthermore, pretreatment of cells with dynasore completely inhibited Rac1 glucosylation by TcdA (Fig 2B and 2C), supporting our earlier observation that TcdA intoxication is dynamin-dependent.

To determine what step in the toxin pathway the inhibitor was affecting, we performed time-of-addition assays. Dynasore was added prior to intoxication (pretreatment), at the same time as toxin (0 min), or at various times post-intoxication, and Rac1 glucosylation in cells by TcdB (S5A and S5B Fig) and TcdA (S5C and S5D Fig) was measured. Results from these experiments show that inhibition of toxin-induced glucosylation can be bypassed by adding dynasore 5 to 10 min post-intoxication, suggesting that the inhibitor is acting at the stage of toxin entry. It is important to note that it takes several minutes for dynasore to appreciably inhibit dynamin-dependent pathways [40], which might explain the glucosylation occurring when the inhibitor and toxin are added together. In summary, our data indicate that TcdA entry and intoxication in epithelial cells require functional dynamin.

### Depletion of caveolin1, cavin1 or PACSIN2 inhibits TcdA-induced toxicity in Caco-2 cells

The above findings indicated that TcdA uptake occurs through a clathrin-independent and dynamin-dependent endocytic mechanism. Dynamin has been implicated or shown to be involved in several CIE pathways such as caveolar endocytosis, the RhoA-dependent pathway, flotillin-dependent endocytosis and endophilinA2-mediated endocytosis (FEME: fast endophilin-mediated endocytosis) [38, 39, 41, 42]. To rapidly assess which of these pathway(s), if any, contribute to TcdA uptake, we performed siRNA-mediated depletion of a panel of host factors involved in these uptake mechanisms and examined their impact on TcdA-induced cell death.

**Fig 3:**
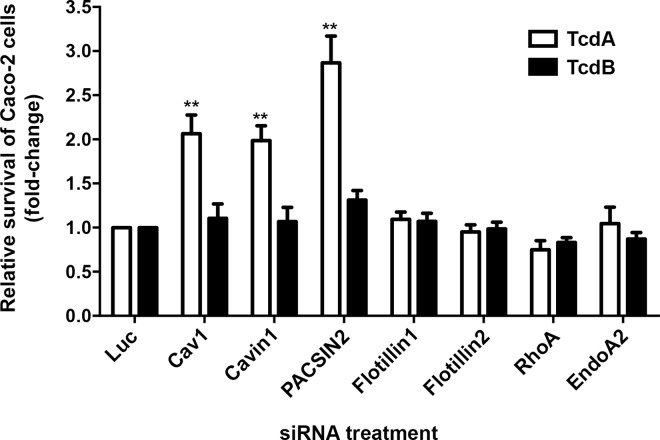
Depletion of caveolin1, cavin1 or PACSIN2 inhibits TcdA-induced toxicity in Caco-2 cells. Caco-2 cells were transfected with 10 nM siRNA against indicated endocytic host factors, exposed to 50 nM TcdA (white bars) or TcdB (black bars) and then assayed for cellular viability using CellTiterGLO. Fold change of survival was obtained by normalizing the relative viability of samples to luciferase control. The data represent the average of at least three independent experiments performed in triplicate with the standard error of the mean indicated as error bars. Data were analyzed using one-way ANOVA. p-values were generated using Dunnett’s multiple comparisons test in GraphPad Prism. **p<0.005. The raw viability data for each siRNA target are provided in S7 Fig.

Caveolae-mediated endocytosis is a commonly studied clathrin-independent and dynamin-dependent pathway [38, 43]. However, there are conflicting reports regarding the expression of caveolin1 (Cav1) in Caco-2 cells [44–46]. Cav1 is typically expressed as two isoforms, Cav1α and Cav1β, and both isoforms can be found in caveolae [47]. Western blotting and RT-PCR analyses show that Caco-2 cells preferentially express the beta isoform of Cav1 (S6 Fig). Since Caco-2 cells express Cav1, we decided to include host factors from the caveolar pathway, namely Cav1, cavin1 and PACSIN2 (protein kinase C and casein kinase substrate in neurons 2), in our siRNA panel. Results from our siRNA screen show that depletion of flotillin1 or 2 (flotillin-dependent pathway), RhoA (RhoA-dependent pathway) or endophilinA2 (endoA2; FEME pathway) does not affect TcdA-induced cytotoxicity in Caco-2 cells. However, knockdown of Cav1, cavin1 or PACSIN2 protected cells from TcdA challenge (Fig 3, S7 and S8 Figs). This protective effect was not observed for cells treated with TcdB, which enters via CME and was used as a negative control.

### TcdA uptake is PACSIN2-dependent but occurs independent of caveolae-mediated endocytosis

Cav1, cavin1 and PACSIN2 are host proteins involved in caveolae-mediated endocytosis [48–52]. Results from our siRNA screen, therefore, lead to the hypothesis that TcdA uptake in Caco-2 cells is mediated by caveolae-dependent endocytosis. However, Vogel *et al*. had previously shown that Caco-2 cells contain extremely few, if any, caveolae [46]. Consistent with their finding, we observed that the cytoplasmic staining of cavin1 in Caco-2 cells was diffuse and atypical of caveolae-associated pools (S9A Fig). Caco-2 cells appear to lack caveolae despite expressing Cav1β and cavin1. Furthermore, we did not observe appreciable colocalization of TcdA-546 with Cav1 or cavin1 in Caco-2 cells (S9A Fig). To better understand the contribution of caveolae-mediated endocytosis to TcdA uptake, we decided to investigate TcdA entry in mouse embryonic fibroblast (MEF) cells, which are sensitive to TcdA and contain caveolae. Confocal microscopy revealed no detectable colocalization of TcdA-546 with Cav1 or cavin1 in MEFs (S9B Fig). Furthermore, investigation of TcdA-induced cell rounding in wildtype and Cav1-/- MEF cells shows that Cav1 is not required for the TcdA cytopathic mechanism (S9C and S9D Fig; S1 and S2 Videos). A similar observation was made using a transient knockdown of Cav1 in wildtype MEF cells (S10A and S10B Fig). Taken together, our data suggest that TcdA uptake in MEFs is caveolae-independent. To test this directly, we measured toxin uptake in MEFs depleted of Cav1. MEF cells were transfected with control (luciferase) or Cav1 siRNA and allowed to internalize TcdA-546. Cav1 and TcdA-546 fluorescence intensities in cells were measured and compared between the two conditions to determine knockdown efficiency and extent of toxin uptake. We found that MEFs transfected with Cav1 siRNA showed a 67% decrease in Cav1 fluorescence staining compared to controls (S10C and S10D Fig). The toxin levels in cells remained unaffected by Cav1 depletion, however, supporting the idea of a caveolin-independent uptake mechanism for TcdA in MEFs (S10C and S10E Fig). In summary, our data from both Caco-2 and MEF cells show that caveolae-mediated endocytosis does not contribute to cellular uptake of TcdA.

**Fig 4:**
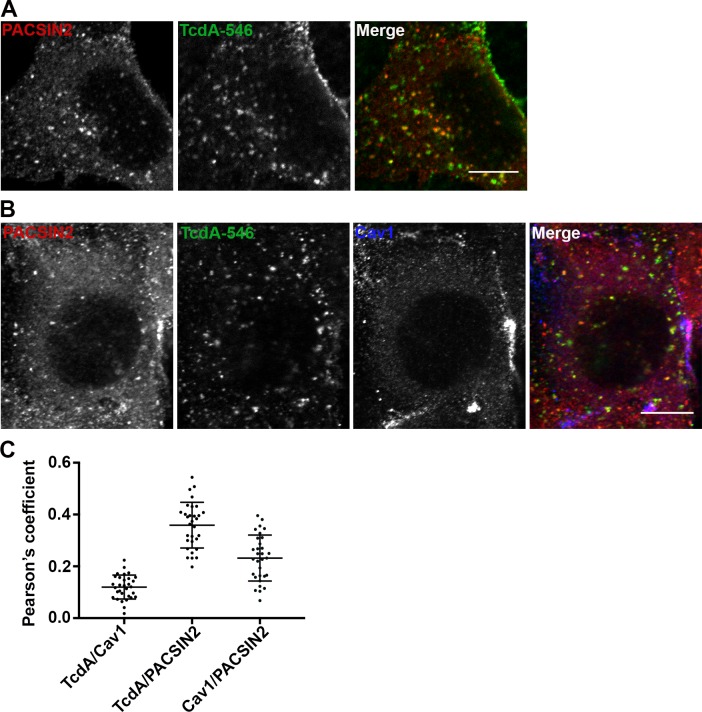
TcdA colocalizes with PACSIN2 in wildtype MEF cells. (**A**) Wildtype MEFs on glass coverslips were allowed to bind 50 nM TcdA-546 for 45 min at 10°C, and cells were shifted to 37°C to allow internalization of toxin for 3 min. Cells were fixed, stained for PACSIN2, and analyzed by confocal microscopy. Merged images show PACSIN2 in red, toxin in green, and colocalization in yellow. Scale bars, 10 μm. The images shown are representative of multiple fields imaged from two independent experiments. (**B**) Immunofluorescence assays were performed as described in (A), but cells were stained for cav1 in addition to PACSIN2. Merged images show PACSIN2 in red, toxin in green, and cav1 in blue. Yellow puncta in merged images denote TcdA- and PACSIN2-positive structures. Pink punta denote caveolae-associated PACSIN2. Scale bars, 10 μm. (**C**) Pearson’s correlation coefficient to assess the extent of colocalization between PACSIN2, cav1 and TcdA-546 after 3 min toxin uptake. Data represent mean and SD of 31 individual cells chosen at random.

**Fig 5:**
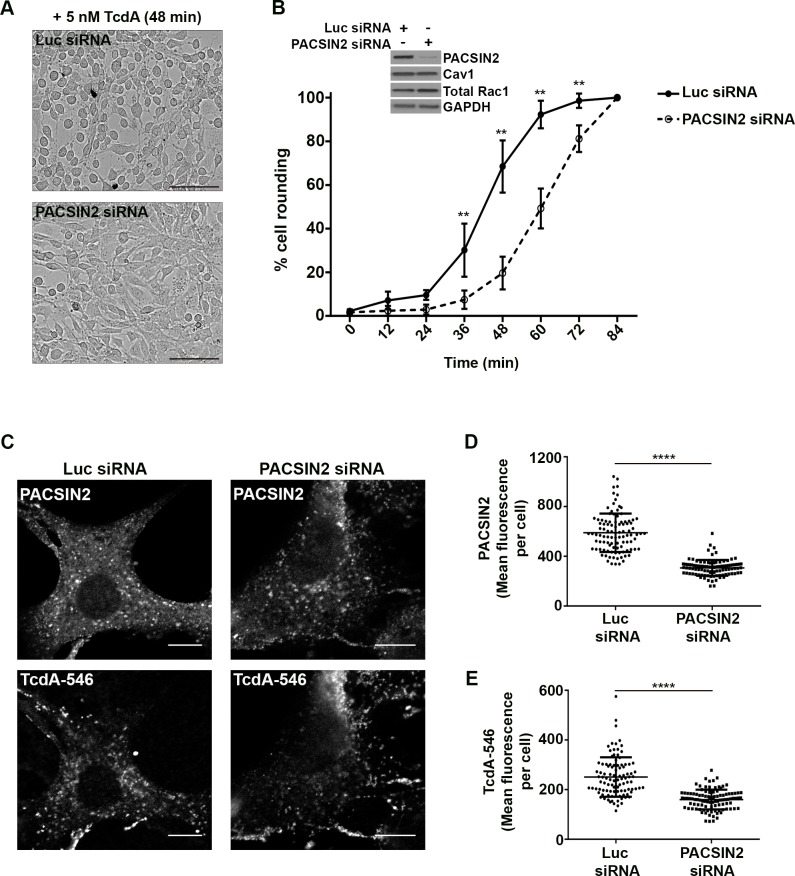
PACSIN2 is required for TcdA uptake and toxin-induced rounding in wildtype MEF cells. **(A) and (B) Depletion of PACSIN2 delays TcdA-induced cell rounding in wildtype MEF cells.** MEF cells transfected with luciferase (non-targeting) or PACSIN2 siRNA were challenged with 5 nM TcdA, and toxin-induced cell rounding effects were monitored using an imaging-based kinetic assay as described in Materials and Methods. Representative images of cells 48 min post-toxin treatment are shown in **(A)**. Scale bars, 100 μm. **(B)** The percentage of rounded cells in each siRNA condition was quantified for the indicated time points. Data represent mean and SD of at least 1200 cells from three independent experiments. Data were analyzed using two-way ANOVA and p-values were generated using Sidak’s multiple comparisons test in GraphPad Prism. **p<0.005. Western blots of whole cell lysates shown in inset indicate that PACSIN2 siRNA transfection resulted in a significant decrease (96.3 ± 2.8%) in PACSIN2 protein levels but did not affect Cav1 or Rac1 levels in cells. **(C), (D) and (E). Depletion of PACSIN2 reduces TcdA uptake in MEF cells.** Wildtype MEF cells expressing luciferase (luc) or PACSIN2 siRNA were incubated with 50 nM TcdA-546 at 10°C for 45 min. Cells were allowed to warm up to 37°C for 2 min and then washed to remove unbound toxins and incubated with fresh media prewarmed to 37°C. Bound toxins were allowed to internalize for 9 min at 37°C. Cells were then fixed, stained for PACSIN2 and imaged by confocal microscopy. PACSIN2 and TcdA-546 staining from each condition are shown in **(C)**. Scale bars, 10 μm. The images shown are representative of multiple fields imaged from two independent experiments. **(D)** Comparison of mean fluorescence intensities of PACSIN2 between luc and PACSIN2 siRNA transfected cells. Data represent mean and SD of 101 individual cells. Student’s t test ***p<0.0001. **(E)** Comparison of mean fluorescence intensities of TcdA-546 between luc and PACSIN2 siRNA transfected cells. Data represent mean and SD of 101 individual cells. Student’s t test ***p<0.0001. Cells were chosen at random for intensity analyses.

Interestingly, despite the lack of significant colocalization with Cav1-positive vesicles, TcdA-546 colocalized with PACSIN2 in wildtype MEF cells (Fig 4). PACSIN2/Syndapin-II is a BAR (Bin/amphiphysin/rvs)-domain-containing protein that has been shown to interact with dynamin and regulators of actin to induce membrane curvature and the formation of vesicular-tubular invaginations that can promote receptor-mediated endocytosis [53–55]. Our cell imaging studies show that there is a pool of PACSIN2 that colocalizes with Cav1-positive vesicles, consistent with the known role of PACSIN2 in caveolar endocytosis (Fig 4B and 4C). However, the PACSIN2 that colocalizes with TcdA-546 in MEF cells is not Cav1-associated (Fig 4B and 4C). We also found that PACSIN2 depletion, unlike that of Cav1, protects wildtype MEF cells from TcdA-induced cytopathic effects (Fig 5A and 5B). To test whether PACSIN2 is involved in TcdA entry, we depleted PACSIN2 in wildtype MEFs by using siRNAs and examined the impact on TcdA binding and uptake. We observe that a 61% reduction in PACSIN2 staining has no impact on the overall toxin binding to cells (S11 Fig). However, a 48% reduction in PACSIN2 fluorescence correlates with a 36% decrease in TcdA uptake (Fig 5C, 5D and 5E). Similar uptake assays performed with transferrin, a clathrin-dependent cargo, show that transferrin uptake is not affected by PACSIN2 depletion, and indicates a specific role for PACSIN2 in TcdA entry (S12 Fig).

**Fig 6:**
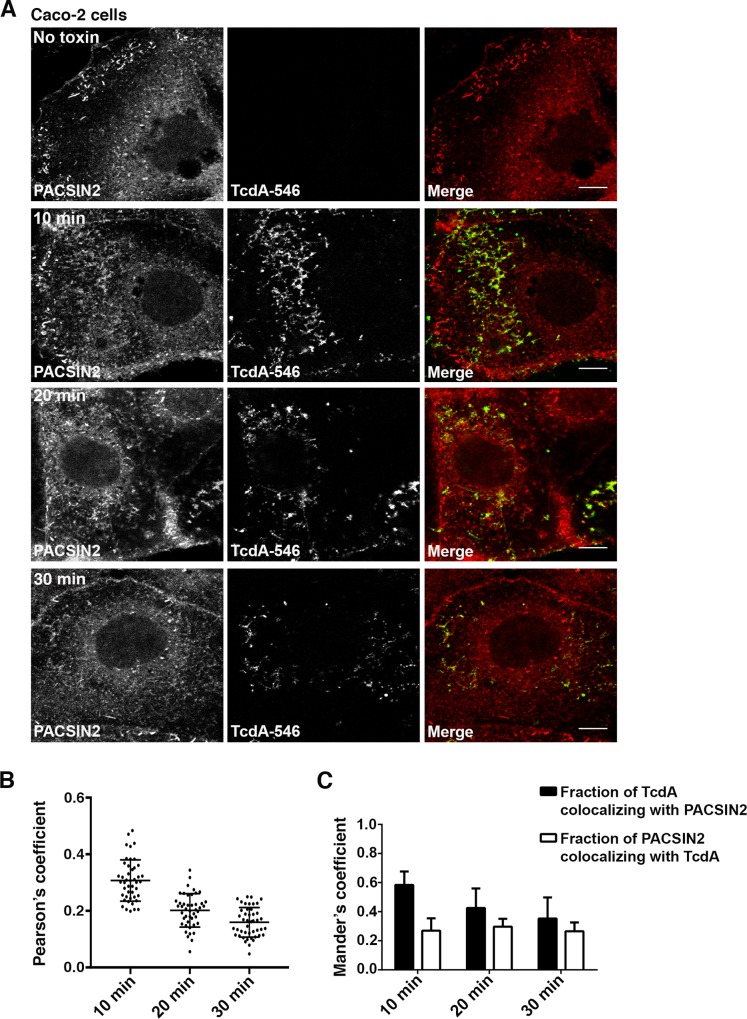
TcdA colocalizes with PACSIN2 during entry in Caco-2 cells. **(A)** Immunofluorescence assays were performed as described in Fig 1B. At indicated time points, cells were fixed, stained for PACSIN2 and analyzed by confocal microscopy. Merged images show PACSIN2 in red, toxin in green and colocalization in yellow. Scale bars, 10 μm. The images shown are representative of multiple fields imaged from three independent experiments (**B**) Pearson’s correlation coefficient to assess the extent of colocalization between PACSIN2 and TcdA-546 at the indicated time points. Data represent mean and SD of 45 individual cells. **(C)** Mander’s coefficient to assess the fraction of TcdA-546 colocalizing with PACSIN2 and vice versa. A value of 1.0 indicates 100% overlap between the two colors. Data represent mean and SD of 45 individual cells. Cells were chosen at random for colocalizaton analyses.

We next checked if PACSIN2 is involved in TcdA uptake in Caco-2 cells, which lack the caveolar pathway. Similar to our findings in MEF cells, we observed significant colocalization of TcdA-546 with PACSIN2-positive structures in Caco-2 cells by confocal microscopy (Fig 6). Unlike MEF cells, where TcdA colocalizes with PACSIN2 at 3 min, colocalization in Caco-2 cells was strongest at 10 min post-switch to 37°C (Fig 6B and 6C). PACSIN2 has been shown to associate with Rac1 on early endosomes [56]. It is possible that the TcdA-containing PACSIN2 structures in Caco-2 cells are early endosomes and that we are capturing colocalization at the stage of toxin translocation, where TcdA can access Rac1. To address this, we performed colocalization studies of TcdA-546 with PACSIN2 and early endosomal antigen 1 (EEA1) in Caco-2 cells. As expected, a fraction of PACSIN2 colocalized with EEA1 (S13 Fig). However, these PACSIN2-positive early endosomes were distinct from the toxin-containing PACSIN2 structures observed at 0, 5, 10 and 15 min post-switch to 37°C (S13 Fig). Taken together, our data show that TcdA-546 colocalizes with a Cav1- and endosome-independent pool of PACSIN2 in Caco-2 cells.

**Fig 7:**
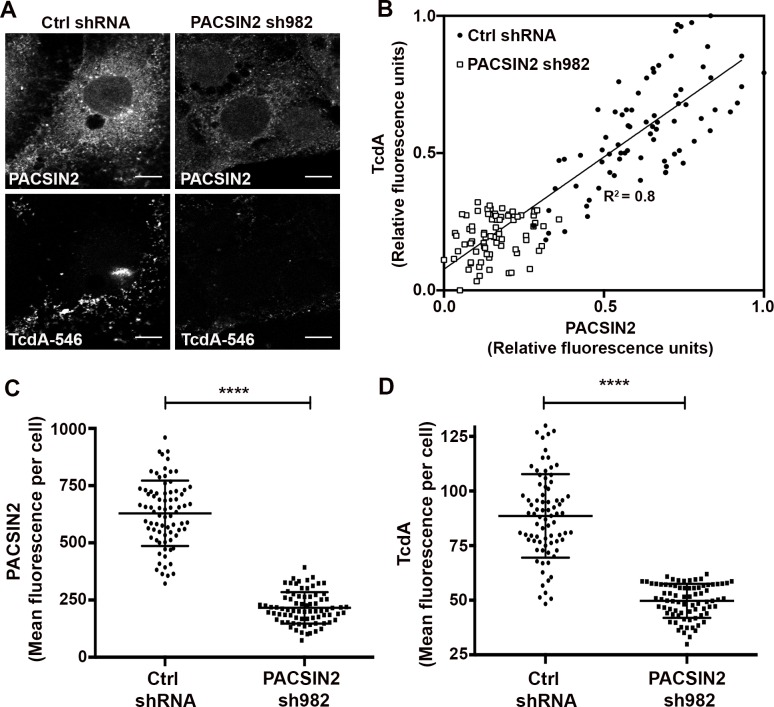
Depletion of PACSIN2 inhibits TcdA entry in Caco-2 cells. **(A)** Caco-2 cells expressing non-targeting shRNA (Ctrl shRNA) or shRNA 982 targeting PACSIN2 were incubated with 50 nM TcdA-546 at 10°C for 45 min. Unbound toxins were removed and cells were shifted to 37°C to allow internalization. After 20 min, cells were washed, fixed, stained for PACSIN2 and imaged by confocal microscopy. PACSIN2 and TcdA staining from ctrl shRNA and sh982 expressing cells are shown. Scale bars, 10 μm. The images shown are representative of multiple fields imaged from three independent experiments **(B)** Scatter plot of the relative fluorescence intensities of PACSIN2 and TcdA in ctrl shRNA (black circles) and PACSIN2 sh982 (white squares) expressing cells. Each data point represents an individual cell. A total of 78 cells per condition were chosen at random for analyses. Linear regression analysis was performed in GraphPad Prism and indicates a strong correlation between PACSIN2 and toxin levels in cells. **(C)** Comparison of mean fluorescence intensities of PACSIN2 between ctrl shRNA and PACSIN2 sh982 expressing cells. Data represent mean and SD of 78 individual cells. Student’s t test ***p<0.0001. **(D)** Comparison of mean fluorescence intensities of TcdA between ctrl shRNA and PACSIN2 sh982 expressing cells. Data represent mean and SEM of 78 individual cells. Student’s t test ***p<0.0001.

Strong colocalization between TcdA-546 and PACSIN2 and inhibition of TcdA-induced cell death upon PACSIN2 depletion (Fig 3 and S14 Fig) suggest that PACSIN2 is required for TcdA entry in Caco-2 cells. To test this, we depleted PACSIN2 and examined the effect on TcdA binding and uptake. Caco-2 cells were transduced with a non-targeting shRNA (ctrl shRNA) and shRNA 982 (sh982) targeting PACSIN2. Expression of sh982 resulted in 94.7 ± 2.1% reduction in PACSIN2 protein levels by western blotting (S15 Fig). Since TcdA binding to cells might be temperature-sensitive [57], we performed binding assays at two different conditions (10°C and 37°C). Irrespective of the temperature, we found that PACSIN2 depletion does not affect TcdA binding to cells (S15 Fig). We then investigated the effect of PACSIN2 depletion on TcdA uptake by imaging-based approaches. Caco-2 cells stably expressing shRNAs were allowed to bind and internalize TcdA-546 and were then stained for PACSIN2 (Fig 7A). Expression of sh982 resulted in a 66% decrease in PACSIN2 fluorescence in cells (Fig 7C). In contrast to control cells, TcdA-546 signal in sh982-expressing cells was typically restricted to the cell periphery suggesting that toxin internalization is inhibited in these cells (Fig 7A). Consistent with that, cells expressing sh982 showed a 44% reduction in TcdA-546 fluorescence compared to cells expressing ctrl shRNA (Fig 7D). Despite similar cell surface binding, the overall levels of cell-associated toxin in PACSIN2-depleted cells were lower than that of controls. PACSIN2 depletion inhibited TcdA entry, and the toxin that is stuck on the outside and unable to enter the cells was likely lost in the subsequent wash steps. We also observed a strong correlation (R^2^ = 0.8) between TcdA-546 and PACSIN2 fluorescence in cells (Fig 7B). Linear regression analyses show that an increase in PACSIN2 fluorescence correlated with a corresponding increase in toxin fluorescence (and vice versa), supporting the conclusion that TcdA uptake is PACSIN2-dependent. Lastly, to evaluate the specificity of our observations with PACSIN2, we performed colocalization and uptake assays in Caco-2 cells with transferrin (a clathrin-dependent cargo). Confocal assays reveal minimal to no colocalization between transferrin and PACSIN2 at 1 min post-switch to 37°C (S16B and S16C Fig). However, the degree of colocalization increased with time. Transferrin has been previously shown to be transported to PACSIN2-positive perinuclear vesicles upon entry [56]. Consistent with that, we found a significant portion of transferrin in PACSIN2-positive endosomes at 3 min post-entry (S16A Fig). This is in contrast to TcdA, which colocalizes with an endosome-independent pool of PACSIN2 in Caco-2 cells (S13 Fig). It is important to note that while transferrin colocalizes with PACSIN2-positive endosomes, the uptake of this cargo in Caco-2 cells does not require PACSIN2 (S17 Fig). In sum, our findings emphasize a specific requirement for PACSIN2 in the TcdA uptake mechanism.

## Discussion

TcdA and TcdB are the key virulence factors that mediate the pathology associated with *C*. *difficile* infection [5, 6]. Cellular intoxication by TcdA and TcdB depends on endocytosis and transport to acidified endosomal compartments within cells [18, 22, 23, 58]. Since these toxins represent excellent targets for therapeutic intervention, understanding the mechanism of toxin entry is a significant priority.

TcdB has been shown to require clathrin-mediated endocytosis (CME) to induce Rac1-inactivation and cell rounding [36]. However, the endocytic mechanisms utilized by TcdA for entry and intoxication have not been clearly defined. In this study, we have combined several independent approaches to obtain a detailed understanding of TcdA entry into cells. We perturbed the function of several key endocytic factors using RNAi-mediated depletion or pharmacological inhibition and determined the subsequent effect on TcdA uptake and toxin-induced downstream effects such as Rac1 glucosylation, cell rounding or cell death. The use of TcdB and transferrin as a controls in these assays allowed for comparative analyses of the endocytic factors (or pathways) relevant for the cytotoxic mechanism of both toxins and for evaluation of the specificity of the perturbations made. Additionally, we validated our findings from the perturbation studies by examining the colocalization of fluorescently labeled TcdA with markers of specific endocytic pathways.

Using these complementary approaches, we find that TcdA uptake in Caco-2 cells is independent of CME. First, both transient and stable depletion of CHC had no effect on TcdA-induced cytotoxicity. Second, TcdA did not colocalize with markers of the clathrin-mediated endocytic pathway. Our results contradict previous reports that propose a role for CME in TcdA uptake [36, 37]. While the first study reported that TcdA uptake occurs via CME [36], the second study has implicated both clathrin-dependent and–independent pathways in TcdA uptake [37]. Both studies relied primarily on pharmacological inhibition of CME by chlorpromazine to investigate the contribution of CME to TcdA entry. Pretreatment of HeLa or HT-29 cells with chlorpromazine was shown to reduce Rac1 glucosylation and cell rounding by TcdA [36, 37]. Consistent with these studies, we observed that chlorpromazine treatment also reduced Rac1 glucosylation by TcdA in Caco-2 cells. While chlorpromazine has been widely used to disrupt clathrin-coated pits, several studies demonstrate that the drug can also interfere with clathrin-independent endocytic mechanisms [41, 59–61]. It is therefore important to corroborate the data obtained by pharmacological inhibition with other more specific approaches. Interestingly, in contrast to their chlorpromazine experiments, Gerhard *et al*. did not observe a significant decrease in Rac1 glucosylation by TcdA in HT-29 cells depleted of CHC [37]. This would argue that TcdA intoxication in HT-29 cells is independent of CME, consistent with our findings in Caco-2 cells.

Clathrin-independent endocytic (CIE) pathways can be subdivided based on whether or not they use a dynamin GTPase for vesicle scission [62, 63]. Macropinocytosis, clathrin-independent carriers (CLIC) and Arf6-regulated pathways are dynamin-independent, whereas dynamin has been implicated or shown to be involved in caveolae-, RhoA-, flotillin- and endophilinA2-mediated endocytosis [38, 39, 41, 62, 63]. Results from our perturbation studies indicate that TcdA internalization is dynamin-dependent. Using a siRNA-based screen of endocytic factors from dynamin-dependent pathways, we were able to identify Cav1, cavin1 and PACSIN2 to be important for TcdA-mediated cell death.

Cav1, cavin1 and PACSIN2 are key proteins involved in caveolae formation and endocytosis. Cav1 is a major structural component of caveolae membrane coats [51]; disruption of Cav1 leads to loss of caveolae [48], and ectopic expression of Cav1 in cells lacking caveolae results in *de novo* formation of caveolae [64]. Cavin1 or PTRF (polymerase I transcript release factor) is a caveolae-associated protein that is required for the formation of caveolae via sequestration of caveolins into caveolae [50]. PACSIN2/syndapin-II is a Fer-CIP4 homology-BAR (F-BAR) domain-containing protein that is involved in the membrane sculpting of caveolae and recruitment of dynamin for caveolae fission [49, 52]. We found no evidence for a direct involvement of caveolae-mediated endocytosis in TcdA uptake. Imaging studies in Caco-2 cells showed no detectable colocalization between TcdA and Cav1 or cavin1. However, we made two observations worth noting. First, Caco-2 cells do not express the α isoform of Cav1. Fujimoto *et al*. reported that expression of the Cav1 α isoform, but not β isoform, resulted in the formation of caveolar invaginations in cells that lack endogenous caveolae, suggesting that the α isoform is required for functional caveolae formation [47]. Second, we observed a strong nuclear but diffuse cytoplasmic staining for cavin1 in Caco-2 cells. These observations are in line with a previous report by Vogel *et al*. [46], which showed that Caco-2 cells lack functional caveolae. Lack of Cav1α and functional caveolae likely affects the localization and function of cavin1 in these cells resulting in the atypical staining pattern. We also did not observe colocalization between TcdA and Cav1 or cavin1 in mouse embryonic fibroblast cells that do contain functional caveolae. Furthermore, depletion of Cav1 does not affect TcdA uptake or TcdA-induced cytopathic effects in MEFs indicating that TcdA uptake can occur independent of caveolae-mediated endocytosis.

We speculate that Cav1 and cavin1 promote TcdA-induced toxicity in Caco-2 cells through indirect mechanisms. One possibility is that these proteins regulate the expression or function of endocytic factors involved in the TcdA uptake mechanism. There is emerging evidence for such crosstalk between caveolar proteins and other CIE pathways [65, 66]. Additionally, Cav1 and cavin1 are involved in cholesterol trafficking and homeostasis [67–69]. TcdA requires cholesterol for pore-formation and toxicity [70]. Depletion of caveolar proteins may modulate the lipid composition of cell membranes leading to indirect effects on TcdA toxicity. However, Cav1 depletion does not affect TcdA-induced cytopathic effects in MEF cells, making this unlikely to be the mechanism involved. Finally, caveolar proteins could be involved in signaling mechanisms or recycling of receptors that promote TcdA-induced toxicity. We currently do not know the receptor(s) or the exact uptake mechanism for TcdA in Caco-2 and MEF cells. Therefore, it is difficult to determine which of these indirect mechanisms, if any, contribute to the effects observed in our siRNA-viability assay.

Interestingly, we find that TcdA uptake, while caveolae-independent, is dependent on PACSIN2. In MEF cells, which contain caveolae, TcdA colocalizes with PACSIN2, and depletion of PACSIN2 inhibits TcdA entry and toxin-induced downstream effects. We made similar observations in Caco-2 cells, which lack caveolae. Proteins in the PACSIN/syndapin family have an N-terminal F-BAR domain that mediates F-actin binding and membrane bending and a C-terminal Src homology 3 (SH3) domain that can interact with dynamin, synaptojanin and Neuronal Wiskott-Aldrich Syndrome Protein (N-WASP), a component of the actin polymerization machinery [53, 71–75]. PACSIN2 is ubiquitously expressed, whereas PACSIN1 is mainly expressed in brain, and PACSIN3 is expressed predominantly in skeletal muscles, lung and heart [73, 76, 77]. PACSINs form homo- and hetero- oligomers that allow them to function as adapter or scaffolding proteins that can link the actin cytoskeleton with the endocytic machinery [54, 55, 78]. Previously, PACSIN2 has been shown to be involved in epidermal growth factor receptor (EGFR) internalization and cholera toxin B (CTxB) entry [52, 56, 79]. Similar to our observations with TcdA, CTxB has been shown to colocalize with PACSIN2 in HeLa cells [56], and depletion of PACSIN2 results in a significant decrease in CTxB incorporation into HeLa cells [52]. It is not clear from these studies however, whether PACSIN2 functions independently of caveolae-mediated endocytosis in promoting CTxB entry.

While PACSIN2 is required for TcdA uptake in both Caco-2 and wildtype MEF cells, the molecular and mechanistic details of this PACSIN2-dependent uptake may differ between these cells. In wildtype MEF cells, our images show TcdA in PACSIN2-positive structures that have a vesicular appearance, whereas in Caco-2 cells, we observe TcdA in PACSIN2 structures with different curvature. PACSIN2 is a BAR-domain protein that bends membranes. In addition to being associated with vesicular structures such as caveolae, PACSIN2 is also known to create tubular membrane invaginations [53]. We speculate that in Caco-2 cells, TcdA is internalized into small tubules or tubular constrictions induced by PACSIN2, resulting in the extended structures we observe in our images. We do not know why toxin is internalized into PACSIN2 structures with different curvatures in Caco-2 vs MEF cells, and this is an area for future investigation.

Overall, our data indicate that in Caco-2 and MEF cells TcdA uptake and intoxication occurs by a clathrin- and caveolae-independent endocytic mechanism that requires PACSIN2. While our work supports an important role for PACSIN2 and dynamin in TcdA uptake and cytotoxicity, we cannot conclude that TcdA entry occurs solely by this mechanism. Alternate routes of entry may exist for TcdA, but based on our perturbation studies, we anticipate that their contribution will be minor. Our work also shows that TcdA and TcdB utilize distinct endocytic pathways to intoxicate epithelial cells. TcdA and TcdB bind different cell surface proteins and sugars [19, 31–33, 35], which likely explains their internalization by distinct endocytic pathways. Importantly, the differences in entry between TcdA and TcdB can have implications regarding their cytotoxic mechanisms. TcdB is more than potent than TcdA in cell culture and animal models [5, 80, 81]. TcdB causes necrosis and extensive damage to the colonic epithelium by inducing the production of reactive oxygen species (ROS). ROS generation by TcdB requires internalization of TcdB-receptor complexes and the activated NADPH oxidase complex via CME and the subsequent formation of a redox active endosome [30]. TcdA, however, is unable to induce ROS [30]. We speculate that the clathrin-independent, PACSIN2-dependent entry mechanism utilized by TcdA prevents the assembly of the redox active endosomes, resulting in reduced toxicity compared to TcdB.

Many aspects of this PACSIN2- and dynamin-dependent endocytic mechanism remain to be elucidated, including how PACSIN2 mediates vesicle formation. Recently, Boucrot *et al*. and Renard *et al*. described a new endocytic route (FEME pathway) mediated by endophilinA2, which is a BAR domain-containing protein similar to PACSIN2 [41, 42]. The FEME pathway is a clathrin-independent and dynamin-dependent pathway that mediates internalization of various clathrin-independent cargoes including Shiga and cholera toxins [41, 42]. Binding to cargo receptor and recruitment of dynamin is mediated by the SH3 domain of endophilin, membrane curvature is induced by the BAR domain, and membrane scission is achieved by the cooperative actions of endophilin, actin and dynamin [41, 42, 82]. We speculate that PACSIN2 can mediate vesicle formation and release in a manner similar to that of endophilin. We also do not know how TcdA is able to gain entry by this pathway and what other host proteins in addition to PACSIN2 and dynamin are required for this process. For future studies, it will be important to identify the TcdA receptor and characterize the toxin-receptor interactions that are necessary for entry by this pathway. We hope to use TcdA as a tool to screen for host proteins that play a role in this pathway.

In conclusion, our study identifies an important route of entry for TcdA in cells that could be targeted for therapeutic purposes, and expands our understanding of PACSIN2’s role in endocytosis. In the future, it will be important to investigate how this PACSIN2 pathway is regulated and if this is a generalized mechanism that TcdA can utilize in cell types other than Caco-2 and MEF cells.

## Materials and Methods

### Cell culture

Caco-2 cells (ATCC HTB-37) were maintained in Minimum Essential Medium (MEM) supplemented with 10% fetal bovine serum (FBS; Atlanta Biologicals), 1% MEM non-essential amino acids (M7145; Sigma), 1% Hepes buffer (15630080; Gibco) and 1% sodium pyruvate (S8636; Sigma). HeLa cells (ATCC CCL-2), HEK 293T cells (ATCC CRL-11268), wildtype (ATCC CRL-2752) and caveolin1^-/-^ mouse embryonic fibroblast (MEF) cells (ATCC CRL-2753) were grown in Dulbecco’s Modified Eagle’s Medium (DMEM) supplemented with 10% FBS. Dynasore (D7693; Sigma) was dissolved in DMSO to obtain a 25 mM stock and was used at a final concentration of 80 μM. Dynasore experiments were performed under serum-free media conditions as the inhibitor binds to serum proteins and loses activity [83].

### Toxin expression and purification

Plasmids encoding wildtype TcdA and TcdB were transformed into *Bacillus megaterium* according to the manufacturer’s instructions (MoBiTec). Recombinant toxins were expressed and purified as described previously with some modifications [84]. *B*. *megaterium* expression strains were grown in LB containing 10 mg/L tetracycline and 35 mL overnight culture was used to inoculate 1 L of media. Bacteria were grown at 37°C with shaking at 220 rpm. Toxin expression was induced with 5 g D-xylose once the culture reached OD_600_ = 0.5. Cells were harvested after 4 h and resuspended in 200 mL of binding buffer (20 mM Tris [pH 8.0], 100 mM NaCl for TcdA and 20 mM Tris [pH 8.0], 500 mM NaCl for TcdB) supplemented with DNase, 400 μL of lysozyme (10 mg/mL) and protease inhibitors (P8849; Sigma). Cells were lysed using an Emulsiflex homogenizer, and lysates were centrifuged at 48,000 g for 30 min. The proteins were purified from the supernatant by Ni-affinity, anion exchange and size exclusion chromatography. Toxins were eluted and stored in 20 mM HEPES (pH 7.0), 50 mM NaCl.

### Viability assays

Caco-2 cells were seeded at a density of 1,000 cells per well in a 384-well plate and incubated at 37°C for 48 h. Cells were then challenged with serial dilutions of TcdA (unlabeled or alexa546 labeled) or TcdB in triplicate. ATP levels of TcdB- and TcdA-treated cells were quantified 24 h and 48 h post intoxication, respectively, by addition of CellTiter-Glo (G7571; Promega) and used as a measure of cellular viability. Luminescence was read using a BioTek Synergy 4 plate reader. Relative cell survival was determined by normalizing the ATP levels of toxin-treated cells to untreated controls.

### siRNA assays

For viability assays, Caco-2 cells (1000 cells/well) were reverse-transfected with 10 nM siRNA against luciferase (non-targeting; negative control) or various targets (Thermo Fisher Scientific) using RNAiMax transfection reagent (13778075; Thermo Fisher Scientific) as described previously [30]. Transfections were performed in a 384-well plate format, with 8 wells per target and toxin treatment. Three wells received mock treatment and three wells received 50 nM of TcdA for 48 h or 50 nM TcdB for 24 h and viability was assayed using CellTiter-Glo. Cells from the remaining two wells were collected and used for RNA isolation and RT-PCR analyses. Relative cell survival was determined by normalizing the ATP levels of toxin-treated cells to untreated controls (which is at a value of 1.0). In some instances, cytotoxicity data are represented as fold change of survival, which was obtained by normalizing the relative viability for each target to that of luciferase control. For immunofluorescence assays, wildtype MEF cells (14,000 cells/well) were reverse-transfected with 20 nM siRNA against luciferase, Cav1 or PACSIN2 (Thermo Fisher Scientific) using lipofectamine RNAiMax (1.5 μL per well) as described by the manufacturer. Cells were seeded on 12 mm glass coverslips (# 1.5; Fisherbrand) in 24-well plates and incubated at 37°C for 48 h to achieve sufficient knockdown. Transfected cells were subsequently used for toxin binding and uptake analyses.

### Lentivirus production and transduction of Caco-2 cells

Non-targeting control shRNA (RHS4346) and shRNAs targeting sequences in clathrin heavy chain (V2LHS_67887 and V3LHS_359489) were purchased from GE Healthcare Dharmacon, Inc. PACSIN2 shRNA (TRCN0000037982; Sigma) was a gift from Matt Tyska (Vanderbilt University, Nashville, TN). The packaging plasmids ΔR8.91 and pCMVG were a kind donation from Chris Aiken (Vanderbilt University Medical Center, Nashville, TN). For stable knockdowns, shRNA plasmids were packaged into lentiviral particles for transduction. Briefly, HEK293T cells were plated in a 10 cm dish and transfected with 1 mL of serum free media containing 30 μg total DNA (15 μg shRNA plasmid + 11.25 μg ΔR8.91 + 3.75 μg pCMVG) preincubated with 90 μL of 1 mg/mL of PEI. After 48 h of transfection, a total of 10 mL media containing virus particles was collected and passed through a 0.45 μm filter and stored in 1 mL aliquots at– 80°C. Caco-2 cells were plated in 75- cm2 flasks such that they were 60% confluent on the day of transduction. Cells were incubated with 1.5 mL of virus supernatant diluted in 3 mL of conditioned media containing 4 μg/mL of polybrene (107689; Sigma) at 37°C for 4 h, then supplemented with 5 mL of conditioned media and incubated overnight. Infected cells were passaged and allowed to recover for 2 days. Transduced Caco-2 cells were then selected by culturing in media containing 10 μg/mL puromycin (P8833; Sigma) for 96 h. To confirm knockdown, whole cell lysates were probed with antibodies against the target protein and GAPDH (loading control).

### RT-PCR analyses

Total RNA was extracted using the RNeasy Mini Kit (74104; Qiagen). Target mRNAs were amplified from 10 ng of template RNA using a OneStep RT-PCR kit (210212; Qiagen). Primers used are listed in S1 Table. GAPDH mRNA was amplified as a loading control. The RT-PCR products were resolved on a 1–1.5% agarose gel and imaged using the KODAK EDAS 290 digital camera system.

### Cell binding assays

Caco-2 cells expressing ctrl shRNA and PACSIN2 sh982 were seeded at a density of 400,000 cells/well in a 6-well plate format and incubated at 37°C for 48 h. For the binding assay, cells were switched to 10°C for 1 h and then intoxicated with 30 nM TcdA. Toxins were allowed to bind at 10°C for 1 h. Media containing unbound toxin inoculum were then removed and cells were washed twice with ice cold PBS. Cells were dislodged by using a cell scraper, collected and pelleted at 1000 g for 5 min. Cell pellets were homogenized to obtain lysates for SDS PAGE and Western blot. For assays performed at 37°C, cells were initially incubated with toxin suspension for 30 min at 10°C and then switched to 37°C for 4 min. The brief incubation warms the cells to 37°C but does not allow for appreciable toxin internalization. Cells were then washed twice with PBS prewarmed to 37°C to remove unbound toxins and collected for lysis and western blotting. The blot was probed with antibodies against TcdA, PACSIN2, unglucosylated Rac1, total Rac1 and GAPDH. Additionally, cells that did not receive any toxin were used as control.

### Western blotting

To prepare samples for western blotting, cell pellets were suspended in 60 μL of lysis buffer (10mM Tris-Cl, pH 7.4, 250 mM sucrose, 3 mM Imidazole) supplemented with protease inhibitor cocktail (1:100, P8340; Sigma) and homogenized by passing 20-times through a 27G needle fitted to a sterile 1 mL syringe. Nuclei and debri were pelleted and removed by spinning at 1500 g for 15 min. Samples were then diluted with Laemmli sample buffer containing 2-mercaptoethanol and heated at 95°C for 5 min. Equal volumes were loaded on a 4–20% Mini-Protean gradient gels (Bio-Rad). Proteins were transferred in Tris-Glycine buffer to PVDF membranes at 100 V for 1 h and blocked with 5% milk in PBS containing 0.1% Tween-20 (PBST) overnight. Primary antibodies against clathrin heavy chain (1:2000, ab21679; Abcam), GAPDH (1:3000, sc-25778; Santa Cruz), unglucosylated Rac1 (1:1000, 610650; BD Biosciences), total Rac1 (1:1000, 05–389; Millipore), caveolin1α (1:2000, sc-894; Santa Cruz), caveolin1α/β (1:1000, 610060; BD Biosciences), PACSIN2 (1:2000, AP8088b; Abgent), TcdA (1:1000, NB600-1066; Novus Biologicals) and tubulin (1:5000, 3873S; Cell Signaling) were diluted in 5% milk-PBST and incubated with the membranes for 2 h at room temperature. Membranes were washed four times with PBST and then incubated with anti-mouse (7076S; Cell Signaling) or anti-rabbit (7074S; Cell signaling) HRP-linked secondary antibodies for 1 h at room temperature (1:2000 for TcdA, PACSIN2, Rac1, caveolin1 and CHC; 1:5000 for GAPDH and tubulin). Membranes were washed four times with PBST and HRP was detected using ECL Western Blotting Substrate (32106; Pierce). Images of film scans were converted to grayscale and cropped using Photoshop (Adobe Systems).

### Fluorescent labeling of toxins

Purified toxins in 20 mM HEPES (pH 7.0), 50 mM NaCl were incubated with a five-fold molar excess (over the cysteines) of thiol-reactive Alexa Fluor dyes (A10258 and A20347; Thermo Fisher Scientific) at room temperature for 2 h in the dark. Excess dye was removed by dialysis overnight at 4°C using slide-A-lyzer dialysis cassettes (66380; Thermo Fisher Scientific). Toxin concentration and degree of labeling were determined according to manufacturer’s instructions and stored at– 80°C for future use.

### Immunofluorescence staining

1x10^4^ cells were seeded on 12 mm glass coverslips (# 1.5; Fisherbrand) in 24-well plates and incubated at 37°C for 48 h. For internalization assays, cells were chilled at 10°C for 45 min and then incubated with media containing 50 nM labeled toxin or buffer (no toxin control) at 10°C for 45 min. Unbound toxins were removed, and cells were switched to 37°C for various time intervals to allow internalization of toxin. Caco-2 cells lose their actin cytoskeleton and begin to round after 30 min of toxin treatment. Therefore, for imaging experiments 30 min was chosen as the last time interval for staining and image analyses. At each time interval, cells were washed once with pre-warmed PBS and fixed with 4% paraformaldehyde in PBS at 37°C for 15 min. Following fixation, cells were quenched with 0.1 M glycine in PBS, washed three times with PBS and permeabilized with 0.2% Triton X-100/PBS for 3 min at room temperature (RT). Cells were washed three times with PBS and blocked overnight at 4°C in PBS containing 4% BSA, 5% normal goat serum (Life technologies), 0.1% Tween-20. The following day, cells were washed once in BSA-PBST (1% BSA, 0.1% Tween-20 in PBS). Primary antibodies anti-CHC (1:1000, ab21679; Abcam), rabbit anti-caveolin1 (1:50 for Caco-2 cells and 1:250 for wildtype MEFs, 610060; BD Biosciences), mouse anti-caveolin1 (1:25 for wildtype MEFs (Fig 4), 610493; BD Biosciences), anti-cavin1 (1:100, ab48824; Abcam), anti-EEA1 (1:250, 610457; BD Biosciences) and anti-PACSIN2 (1:100, AP8088b; Abgent) were diluted in BSA-PBST and incubated with cells for 2 h at RT. Primary antibody was removed and cells were washed four times with BSA-PBST, and incubated with goat anti-rabbit Alexa Fluor 647 (A-21245; Life Technologies), goat anti-mouse Alexa Flour 488 (A-11029; life Technologies) or goat anti-rabbit Alexa Fluor 546 (A-11035; Life Technologies) in BSA-PBST for 1 h at RT (1:1000 for CHC; 1:500 for caveolin1, cavin1, EEA1 and PACSIN2). Cells were washed twice in BSA-PBST and twice in PBS and then mounted using Prolong Gold Antifade Mountant (P36931; Life Technologies). For actin staining, cells were incubated with 1:100 dilution of Phalloidin-647 (A2287; Thermo Fisher Scientific) for 30 min at RT. The phalloidin incubation step was performed after secondary antibody staining and washes. After 30 min incubation, cells were washed twice in BSA-PBST and twice in PBS and then mounted using Prolong Gold Antifade Mountant.

### Confocal microscopy

Slides were imaged using a 63x/ 1.40 numerical aperture (NA) Plan-Apochromat oil immersion objective on a LSM 710 Meta Inverted laser-scanning confocal microscope (Zeiss) located in the Vanderbilt Cell Imaging Shared Resource (CISR) Core. Alexa488 was excited using the 488 nm line of an Argon laser. Alexa546 was excited at 561 nm and Alexa647 was excited at 633 nm using a HeNe laser. Fluorescence emission was detected using filters provided by the manufacturer. Pinhole size was identical for the fluors used. Single sections of 0.49 μm thickness from a Z-stack are presented. For the purpose of presentation, raw images were exported in tiff format and brightness and contrast were adjusted to the same extent using Fiji [85].

### Image analyses and quantification

For measurements of colocalization, individual cells were demarcated and Pearson’s correlation coefficient and Mander’s overlap coefficients were determined using the Colocalization plugin in Fiji. The Mander’s coefficient determines the fraction of channel1 that overlaps with channel2 and vice versa, with 100% overlap resulting in a value of 1. Nuclei were excluded from the colocalization analyses. Qualitative analyses of colocalization were performed using the plot profile feature in Fiji. The pixel intensities along the line were obtained from the original 12-bit image for each channel and plotted as relative intensities over distance using GraphPad Prism. PACSIN2, Cav1, transferrin and TcdA signals in siRNA- or shRNA-expressing cells were determined by demarcating individual cells and measuring the mean fluorescence intensities for each channel using Fiji. The mean fluorescence intensities of PACSIN2 and TcdA (or transferrin) for each cell were converted to relative intensity values and plotted against each other to generate the scatter plot. To determine the correlation between PACSIN2 and TcdA (or transferrin) in cells, a linear regression analysis was performed on the entire data set and R^2^ value for best fit is represented. Cells were chosen at random for colocalization and intensity analyses.

### Cell rounding assay

Wildtype and caveolin1^-/-^ MEF cells were seeded, in triplicate, at 200,000 cells/well in 6-well plates. The following day, cells were chilled at 10°C for 45 min and allowed to bind 10 nM TcdA for 45 min. Plates were then moved to a Cytation 5 Cell Imaging Multi-Mode Reader (BioTek), and bright field images of cell morphology were captured at 15 min intervals for a 5 h time period, using a 20x/ 0.45 NA objective (1220517; BioTek). A total of 4 frames per well were captured at each time point. The chamber was maintained at 37°C with 5% CO_2_ for the duration of this experiment. Real-time videos of cells rounding in response to toxin were generated using Fiji. For quantification, a total of 36 frames from three independent experiments were analyzed for each cell type and results were expressed as the percentage of rounded cells. Cell rounding assays with siRNA transfected cells were performed as described above with a few modifications. Wildtype MEF cells (200,000 cells/well) were reverse-transfected with 20 nM siRNA against luciferase (non-targeting; negative control), Cav1 or PACSIN2 (Thermo Fisher Scientific) using RNAiMax transfection reagent (7 μL per well) as described by the manufacturer. After 48 h, cells were intoxicated with 5 nM TcdA and images were captured every 12 min for a total of 2 h. For quantification, a total of 12 frames (> 100 cells/frame) from three independent experiments were analyzed for each time point and siRNA condition, and results were expressed as the percentage of rounded cells.

### Statistical analyses

Statistical analyses are described in figure legends. A p-value of ≤ 0.05 was considered significant.

## Supporting Information

S1 Fig siRNA-mediated transient depletion of clathrin heavy chain does not affect TcdA-induced cell killing. Caco-2 cells were transfected with 10 nM siRNA against clathrin heavy chain (CHC) or luciferase (Luc; non-targeting control), exposed to 50nM TcdA (white bars) or TcdB (black bars) and then assayed for cellular viability using CellTiterGLO. Fold change of survival was obtained by normalizing the relative viability of samples to luciferase control. The data represent the average of ten independent experiments performed in triplicate with SEM indicated as error bars. Data were analyzed using Welch’s t test. **p<0.005. RT-PCR confirms that siRNA treatment resulted in a decrease in CHC mRNA expression.
(TIF)

S2 Fig Labeling does not affect TcdA function. Caco-2 cells were treated with indicated concentrations of TcdA or TcdA-546 in triplicate. ATP levels were determined using CellTiterGlo and normalized to signal from untreated cells to assess the relative survival of cells post-toxin treatment. Results represent the mean and SEM of three independent experiments. Data were analyzed using two-way ANOVA and p-values were generated using Sidak’s multiple comparisons test in GraphPad Prism. ns, not significant.
(TIF)

S3 Fig Comparison of TcdA fluorescence at 50 nM and 5 nM in Caco-2 cells. **(A)** Caco-2 cells were allowed to bind 50 nM or 5 nM TcdA-546 for 45 min at 10°C. Cells were shifted to 37°C for 4 min, washed, fixed and imaged using a LSM 510 Meta Inverted laser-scanning confocal microscope (Zeiss). In the images, TcdA-546 is shown in green and PACSIN2 in red. Scale bars, 10 μm. **(B)** Comparison of mean fluorescence intensities of TcdA-546 at 50 and 5 nM. Data represent mean and SD of 50 individual cells chosen at random.
(TIF)

S4 Fig TcdB-647 signal intensity in cells is extremely low. Caco-2 cells were allowed to bind 50 nM TcdA-Alexa546 and TcdB-Alexa647 for 45 min at 10°C. Unbound toxins were removed and cells were shifted to 37°C to allow uptake for the times shown. At indicated times, cells were washed, fixed and imaged using a confocal Microscope. 1x merged images on the left show TcdB in red, TcdA in green and DIC in gray. White dotted boxes in the 1x merged images denote areas that were magnified. Scale bars, 20 μm. The profile analyses on the right represent the relative intensity of red and green pixels at each point along the line trace shown in the zoomed color images.
(TIF)

S5 Fig Dynasore time-of-addition assays reveal a block in toxin entry. Rac1 glucosylation assays were performed with 10 nM TcdB (A) or TcdA (C) as described in Fig 2B but the time of addition of dynasore was varied. Dynasore was added 1 h prior to toxin treatment (pretreatment), or at the same time as toxin (0 min post-intox), or at various times post-intoxication. (B) and (D) Three replicates of the experiments shown in panels A and C were quantified by densitometry and represented as the ratio of unglucosylated and total Rac1 levels. Results reflect the mean and SEM, and were analyzed using a one-way ANOVA. p-values were generated using Dunnett’s multiple comparisons test in GraphPad Prism. *p<0.05; **p<0.005; ns, not significant.
(TIF)

S6 Fig Caveolin-1 β isoform is expressed in Caco-2 cells. **(A)** Western blots of whole cell lysates from HeLa and Caco-2 cells probed with antibodies against the α isoform of caveolin-1 (sc-894) and GAPDH. **(B)** Total RNA from HeLa and Caco-2 cells were subjected to RT-PCR analyses to determine the mRNA expression of caveolin-1 transcript variants. GAPDH was amplified as a loading control. **(C)** Western blots of whole cell lysates from Caco-2 and caveolin1^-/-^ mouse embryonic fibroblast (MEF) cells probed with antibodies against caveolin-1 (both isoforms, BD biosciences) and tubulin (loading control).
(TIF)

S7 Fig Depletion of caveolin1, cavin1 or PACSIN2 inhibits TcdA-induced toxicity in Caco-2 cells. Caco-2 cells were transfected with 10 nM siRNA against Cav1 (A), Cavin1 (B), PACSIN2 (C), Flotillin1 (D), Flotillin2 (E), RhoA (F) and EndoA2 (G), exposed to 50 nM TcdA (black bars) or TcdB (gray bars) and then assayed for cellular viability using CellTiterGLO. Relative survival was obtained by normalizing the viability of treated cells to untreated (no toxin) controls. The data represent the average of at least three independent experiments performed in triplicate with the standard error of the mean indicated as error bars. Data were analyzed using t test. *p<0.05.
(TIF)

S8 Fig RT-PCR controls for siRNA-mediated knockdown of endocytic factors. Total RNA from Caco-2 cells transfected with luciferase siRNA (Luc; non-targeting control) and siRNAs targeting various endocytic factors were subjected to RT-PCR analyses. GAPDH was amplified as a loading control. RT-PCR confirms that siRNA treatment resulted in a decrease in target mRNA expression.
(TIF)

S9 Fig TcdA entry does not involve caveolin-mediated endocytosis. **(A) TcdA does not colocalize with caveolin1 (cav1) or cavin1 in Caco-2 cells.** Colocalization studies of TcdA and caveolar endocytic proteins, caveolin1 and cavin1, were performed by binding TcdA-546 to Caco-2 cells at 10°C for 45 min and shifting cells to 37°C to allow toxin uptake. After 10 min, cells were fixed and stained for caveolin1 or cavin1 and imaged using a confocal microscope. Merged images show caveolin1 or cavin1 in red and toxin in green. Scale bars, 10 μm. **(B) TcdA does not colocalize with caveolin1 or cavin1 in wildtype mouse embryonic fibroblast (MEF) cells.** Colocalization studies were performed in wildtype MEFs as described in (A). Toxin internalization occurred at 37°C for 3 min. Scale bars, 10 μm. **(C) Caveolin1** ^**-/-**^ **MEFs are sensitive to TcdA-induced cell rounding.** Wildtype and caveolin1^-/-^ MEFs were challenged with 10 nM TcdA and toxin-induced cell rounding effects were monitored using an imaging-based kinetic assay as described in Materials and Methods. Representative images of cells 0 h and 5 h post-toxin treatment for each cell type are shown. **(D)** The percentage of rounded cells 5 h post-toxin treatment was quantified for each cell type. Data represent mean and SD of at least 900 cells from three independent experiments. Knockout of cav1 was confirmed by probing western blot of whole cell lysates from wildtype and caveolin1^-/-^ MEFs with antibodies against cav1 and GAPDH (loading control) as shown in inset.
(TIF)

S10 Fig Caveolin1 is not required for TcdA uptake and toxin-induced rounding in wildtype MEF cells. **(A) and (B) Depletion of Cav1 does not affect TcdA-induced cell rounding in wildtype MEF cells.** MEF cells transfected with luciferase (non-targeting) or Cav1 siRNA were challenged with 5 nM TcdA, and toxin-induced cell rounding effects were monitored using an imaging-based kinetic assay as described in Materials and Methods. Representative images of cells 48 min post-toxin treatment are shown in **(A)**. Scale bars, 100 μm. **(B)** The percentage of rounded cells in each siRNA condition was quantified for the indicated time points. Data represent mean and SD of at least 1200 cells from three independent experiments. Western blots of whole cell lysates shown in inset confirms that Cav1 siRNA transfection resulted in a significant decrease in Cav1 protein levels (87.9 ± 1.2%) in cells. **(C), (D) and (E). Cav1 depletion does not affect TcdA uptake in MEF cells.** Wildtype MEF cells expressing luciferase (luc) or Cav1 siRNA were incubated with 50 nM TcdA-546 at 10°C for 45 min. Cells were allowed to warm up to 37°C for 2 min and then washed to remove unbound toxins and incubated with fresh media prewarmed to 37°C. Bound toxins were allowed to internalize for 9 min at 37°C. Cells were then fixed, stained for Cav1 and actin, and imaged by confocal microscopy. The images shown in **(C)** are representative of multiple fields imaged from two independent experiments. Merged images show Cav1 in red, TcdA-546 in green and actin (Phalloidin-647) in blue. Scale bars, 10 μm. **(D)** Comparison of mean fluorescence intensities of Cav1 between luc and Cav1 siRNA transfected cells. Data represent mean and SD of 77 individual cells. Student’s t test ***p<0.0001. **(E)** Comparison of mean fluorescence intensities of TcdA-546 between luc and Cav1 siRNA transfected cells. Data represent mean and SD of 77 individual cells and were analyzed by student’s t test. ns, not significant. Cells were chosen at random for intensity analyses.
(TIF)

S11 Fig Depletion of PACSIN2 does not affect TcdA binding in MEF cells. **(A)** Wildtype MEF cells expressing luciferase (luc) or PACSIN2 siRNA were incubated with 50 nM TcdA-546 at 10°C for 45 min. Cells were allowed to warm up to 37°C for 2 min and then washed to remove unbound toxin and fixed. Cells were stained for PACSIN2 and actin (phalloidin-647). The images shown are representative of multiple fields imaged from three independent experiments. Merged images show PACSIN2 in red, TcdA-546 in green, and actin (Phalloidin-647) in blue. Scale bars, 10 μm. **(B)** Comparison of mean fluorescence intensities of PACSIN2 between luc and PACSIN2 siRNA transfected cells. Data represent mean and SD of 95 individual cells. Student’s t test ***p<0.0001. **(C)** Comparison of mean fluorescence intensities of TcdA-546 between luc and PACSIN2 siRNA transfected cells. Data represent mean and SD of 95 individual cells and were analyzed by student’s t test. ns, not significant. Cells were chosen at random for intensity analyses.
(TIF)

S12 Fig Depletion of PACSIN2 does not affect transferrin uptake in MEF cells. **(A)** Wildtype MEF cells expressing luciferase (luc) or PACSIN2 siRNA were incubated with 25 μg/ml of transferrin-alexa546 at 10°C for 45 min. Cells were switched to 37°C for 4 min, fixed and stained for PACSIN2 and actin (phalloidin-647). The images shown are representative of multiple fields imaged from two independent experiments. Merged images show PACSIN2 in red, transferrin-546 in green, and actin (Phalloidin-647) in blue. Scale bars, 10 μm. (B) Comparison of mean fluorescence intensities of PACSIN2 between luc and PACSIN2 siRNA transfected cells. Data represent mean and SD of 76 individual cells. Student’s t test ***p<0.0001. (C) Comparison of mean fluorescence intensities of transferrin-546 between luc and PACSIN2 siRNA transfected cells. Data represent mean and SD of 76 individual cells and were analyzed by student’s t test. ns, not significant. Cells were chosen at random for intensity analyses.
(TIF)

S13 Fig TcdA- and PACSIN2-positive structures at 5, 10 and 15 min post-switch to 37°C are not early endosomes. (**A**) Caco-2 cells on glass coverslips were allowed to bind 50 nM TcdA-546 for 45 min at 10°C. Unbound toxin was removed, and cells were shifted to 37°C to allow internalization of toxin for 0, 5, 10 or 15 min. Cells were fixed, stained for PACSIN2 and early endosomal antigen 1 (EEA1) and analyzed by confocal microscopy. Merged images show PACSIN2 in red, toxin in green and EEA1 in blue. Yellow puncta in merged images denote TcdA- and PACSIN2-positive structures. Pink punta denote PACSIN2-positive endosomes. Scale bars, 20 μm. The arrowheads in the images highlight representative regions that are positive for TcdA and PACSIN2 but not EEA1. The images shown are from the 10 min time point and are representative of multiple fields imaged from two independent experiments. (**B**) Pearson’s correlation coefficient to assess the extent of colocalization between PACSIN2, EEA1 and TcdA-546 at 0, 5, 10 and 15 min post-entry. Data represent mean and SD of 30 individual cells.
(TIF)

S14 Fig Depletion of PACSIN2 inhibits TcdA-induced cell killing at various concentrations tested. **(A)** Caco-2 cells were transfected with 10 nM siRNA against PACSIN2 or luciferase (Luc; non-targeting control) and then intoxicated with indicated concentrations of TcdA or TcdB. ATP levels were determined using CellTiterGlo and normalized to signal from untreated cells to assess the relative survival of cells post-toxin treatment. Results represent the mean and SEM of three independent experiments. Data were analyzed using two-way ANOVA, and p-values were generated using Sidak’s multiple comparisons test in GraphPad Prism. **p<0.005; ns, not significant. **(B)** Western blot of whole cell lysates from siRNA-expressing Caco-2 cells probed with antibodies against PACSIN2, total Rac1 and GAPDH (loading control). PACSIN2 siRNA resulted in 94.4 ± 4.4% reduction in PACSIN2 protein levels by densitometry.
(TIF)

S15 Fig PACSIN2 depletion does not affect TcdA binding to Caco-2 cells. (**A**) Caco-2 monolayers expressing ctrl shRNA and PACSIN2 sh982 were allowed to bind 30 nM TcdA at 10°C. Whole cell lysates were prepared for SDS PAGE and Western blot. The blot was probed with antibodies against TcdA CROPs, PACSIN2, unglucosylated Rac1, total Rac1 and GAPDH. Cells that did not receive any toxin were used as a control. (**B**) Experiments shown in (A) were quantified by densitometry and represented as the ratio of bound toxin and GAPDH levels. Results reflect the mean and SEM of three independent experiments and were analyzed using two-tailed t-test. ns, not significant. **(C)** Experiment was performed as in (A) with some modifications. After toxin binding at 10°C, cells were switched to 37°C for 4 min to warm the cells to 37°C. Cells were then washed with PBS prewarmed to 37°C to remove unbound toxins and collected for lysis and western blotting.
(TIF)

S16 Fig Transferrin colocalizes with PACSIN2-positive endosomes in Caco-2 cells. **(A)** Caco-2 cells on glass coverslips were allowed to bind 25 μg/ml of transferrin-alexa647 for 45 min at 10°C. Cells were shifted to 37°C to allow internalization for 1, 3 and 5 min. Cells were fixed, stained for PACSIN2 and early endosomal antigen 1 (EEA1) and analyzed by confocal microscopy. Merged images show PACSIN2 in red, transferrin in green, and EEA1 in blue. Scale bars, 10 μm. The arrowheads in the images highlight representative regions that are positive for transferrin, PACSIN2 and EEA1. The images shown are from the 3 min time point and are representative of multiple fields imaged from two independent experiments. **(B)** Pearson’s correlation coefficient to assess the extent of colocalization between transferrin and PACSIN2. Data represent mean and SD of at least 43 individual cells. **(C)** Mander’s coefficient to assess the fraction of transferrin colocalizing with PACSIN2 and vice versa. A value of 1.0 indicates 100% overlap between the two colors. Data represent mean and SD of 43 individual cells. Cells were chosen at random for colocalization analyses.
(TIF)

S17 Fig PACSIN2 depletion does not affect transferrin uptake in Caco-2 cells. **(A)** Caco-2 cells expressing non-targeting shRNA (Ctrl shRNA) or shRNA 982 targeting PACSIN2 were incubated with 25 μg/ml transferrin-647 (Tf-647) at 10°C for 45 min. Cells were shifted to 37°C to allow internalization. After 5 min, cells were washed, fixed, stained for PACSIN2 and imaged by confocal microscopy. PACSIN2 and Tf-647 staining from ctrl shRNA and sh982 expressing cells are shown. Scale bars, 10 μm. The images shown are representative of multiple fields imaged from three independent experiments. **(B)** Scatter plot of the relative fluorescence intensities of PACSIN2 and Tf-647 in ctrl shRNA (black circles) and PACSIN2 sh982 (white squares) expressing cells. Each data point represents an individual cell. A total of 72 cells per condition were chosen at random for analyses. Linear regression analysis was performed in GraphPad Prism and indicates a lack of correlation between PACSIN2 and Tf-647 levels in cells. **(C)** Comparison of mean fluorescence intensities of PACSIN2 between ctrl shRNA and PACSIN2 sh982 expressing cells. Data represent mean and SD of 72 individual cells. Student’s t test ***p<0.0001. (D) Comparison of mean fluorescence intensities of Tf-647 between ctrl shRNA and PACSIN2 sh982 expressing cells. Data represent mean and SD of 72 individual cells and were analyzed by student’s t test. ns, not significant.
(TIF)

S1 Table Primers used for RT-PCR analyses. (TIF)

S1 Video Cell rounding induced by TcdA in wildtype MEFs. Wildtype MEF cells were treated with 10 nM TcdA and imaged every 15 min over a 5-hour time period as described in Materials and Methods. A total of 21 frames were compiled to create a real-time video of cells rounding in response to toxin.
(AVI)

S2 Video Cell rounding induced by TcdA in Cav1^-/-^ MEFs. Cav1^-/-^ MEF cells were treated with 10 nM TcdA and imaged every 15 min over a 5-hour time period as described in Materials and Methods. A total of 21 frames were compiled to create a real-time video of cells rounding in response to toxin.
(AVI)
