# Supplementary figures and images for: Clostridium difficile Toxin A Undergoes Clathrin-Independent, PACSIN2-Dependent Endocytosis
Source: PLoS Pathog. 2016 Dec 12;12(12):e1006070. doi: 10.1371/journal.ppat.1006070 (PMC5152916; doi:10.1371/journal.ppat.1006070)

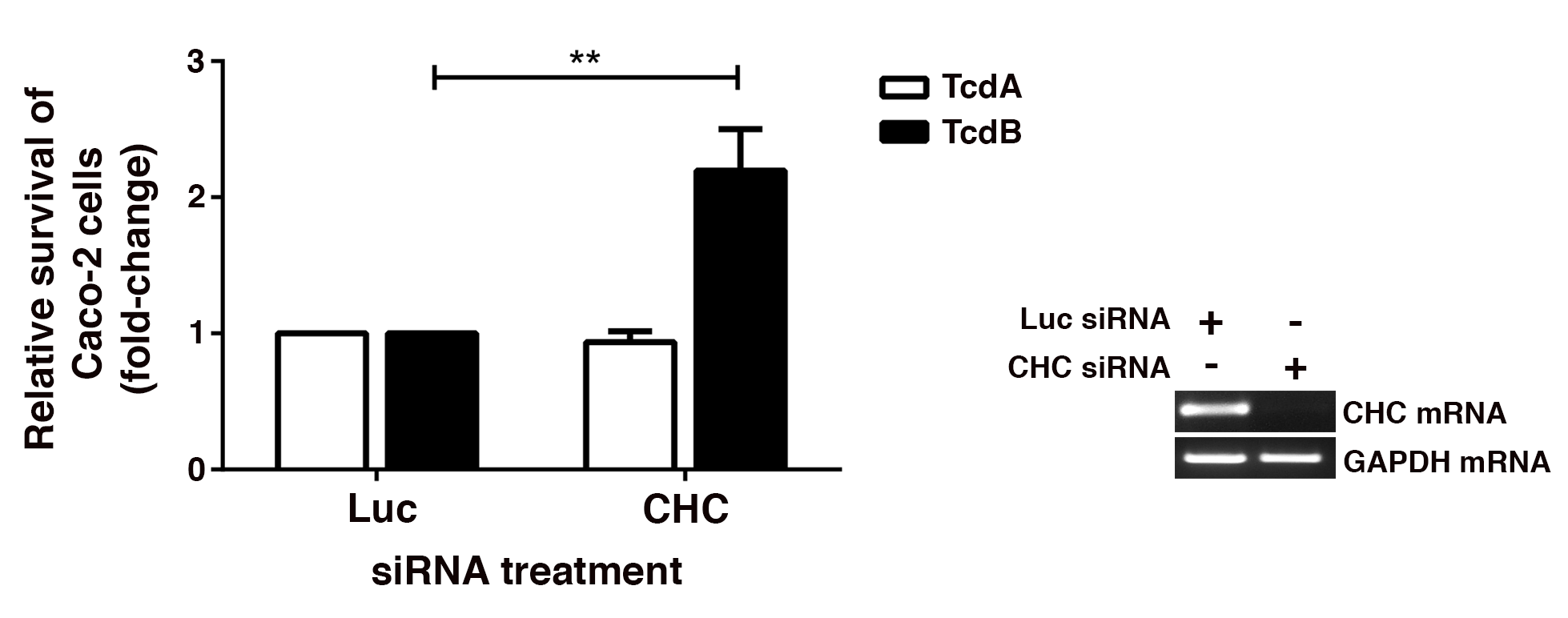

Supplement: S1 Fig — Caco-2 cells were transfected with 10 nM siRNA against clathrin heavy chain (CHC) or luciferase (Luc; non-targeting control), exposed to 50nM TcdA (white bars) or TcdB (black bars) and then assayed for cellular viability using CellTiterGLO. Fold change of survival was obtained by normalizing the relative viability of samples to luciferase control. The data represent the average of ten independent experiments performed in triplicate with SEM indicated as error bars. Data were analyzed using Welch’s t test. **p<0.005. RT-PCR confirms that siRNA treatment resulted in a decrease in CHC mRNA expression. (TIF) [file ppat.1006070.s001.tif]

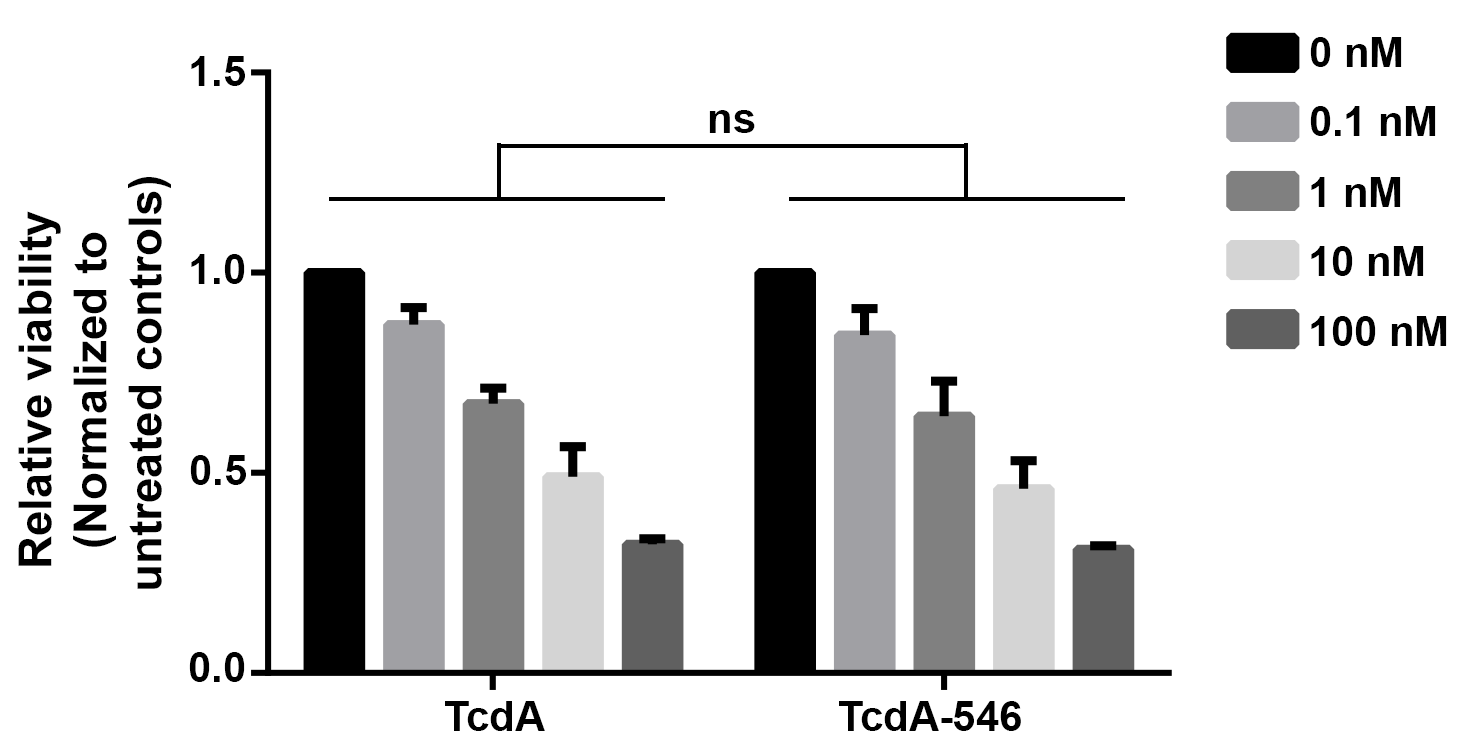

Supplement: S2 Fig — Caco-2 cells were treated with indicated concentrations of TcdA or TcdA-546 in triplicate. ATP levels were determined using CellTiterGlo and normalized to signal from untreated cells to assess the relative survival of cells post-toxin treatment. Results represent the mean and SEM of three independent experiments. Data were analyzed using two-way ANOVA and p-values were generated using Sidak’s multiple comparisons test in GraphPad Prism. ns, not significant. (TIF) [file ppat.1006070.s002.tif]

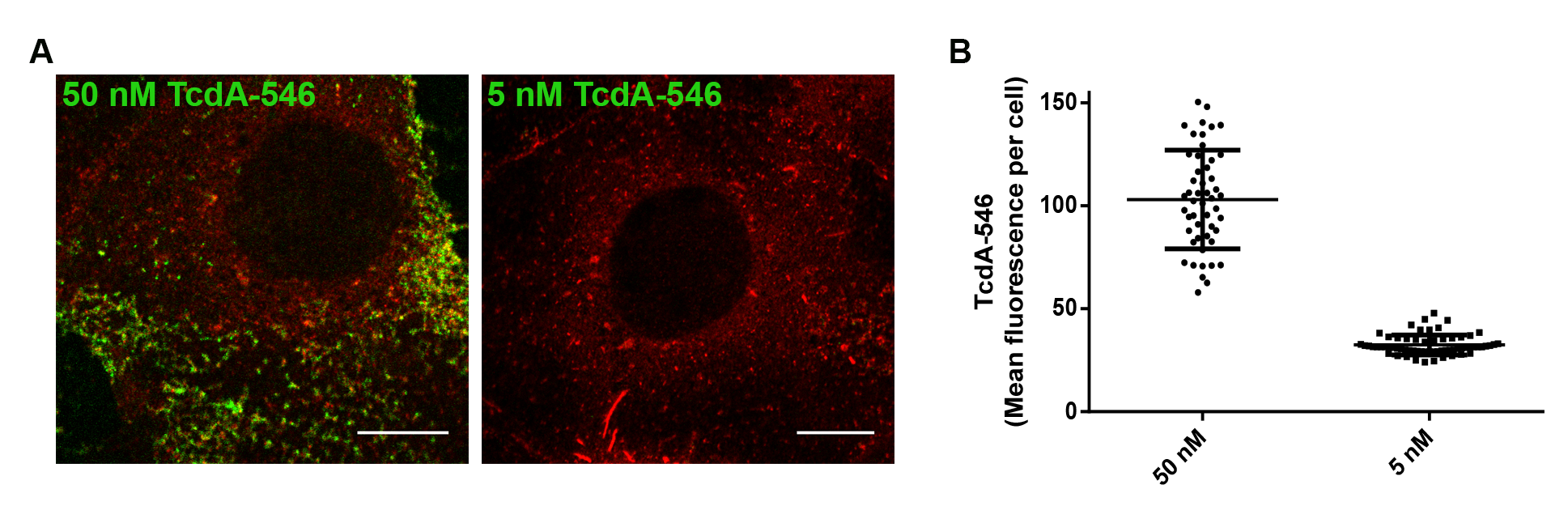

Supplement: S3 Fig — (A) Caco-2 cells were allowed to bind 50 nM or 5 nM TcdA-546 for 45 min at 10°C. Cells were shifted to 37°C for 4 min, washed, fixed and imaged using a LSM 510 Meta Inverted laser-scanning confocal microscope (Zeiss). In the images, TcdA-546 is shown in green and PACSIN2 in red. Scale bars, 10 μm. (B) Comparison of mean fluorescence intensities of TcdA-546 at 50 and 5 nM. Data represent mean and SD of 50 individual cells chosen at random. (TIF) [file ppat.1006070.s003.tif]

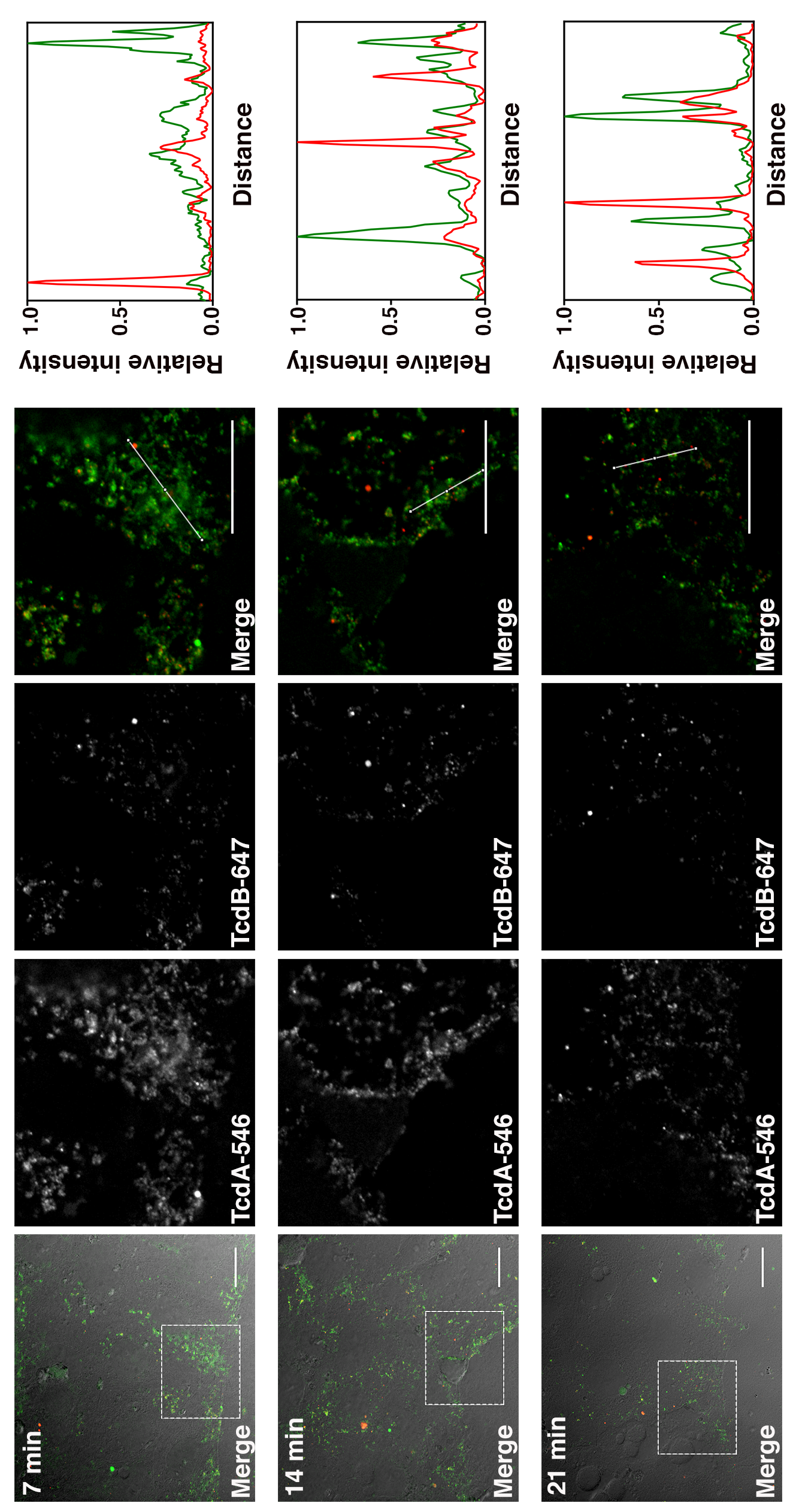

Supplement: S4 Fig — Caco-2 cells were allowed to bind 50 nM TcdA-Alexa546 and TcdB-Alexa647 for 45 min at 10°C. Unbound toxins were removed and cells were shifted to 37°C to allow uptake for the times shown. At indicated times, cells were washed, fixed and imaged using a confocal Microscope. 1x merged images on the left show TcdB in red, TcdA in green and DIC in gray. White dotted boxes in the 1x merged images denote areas that were magnified. Scale bars, 20 μm. The profile analyses on the right represent the relative intensity of red and green pixels at each point along the line trace shown in the zoomed color images. (TIF) [file ppat.1006070.s004.tif]

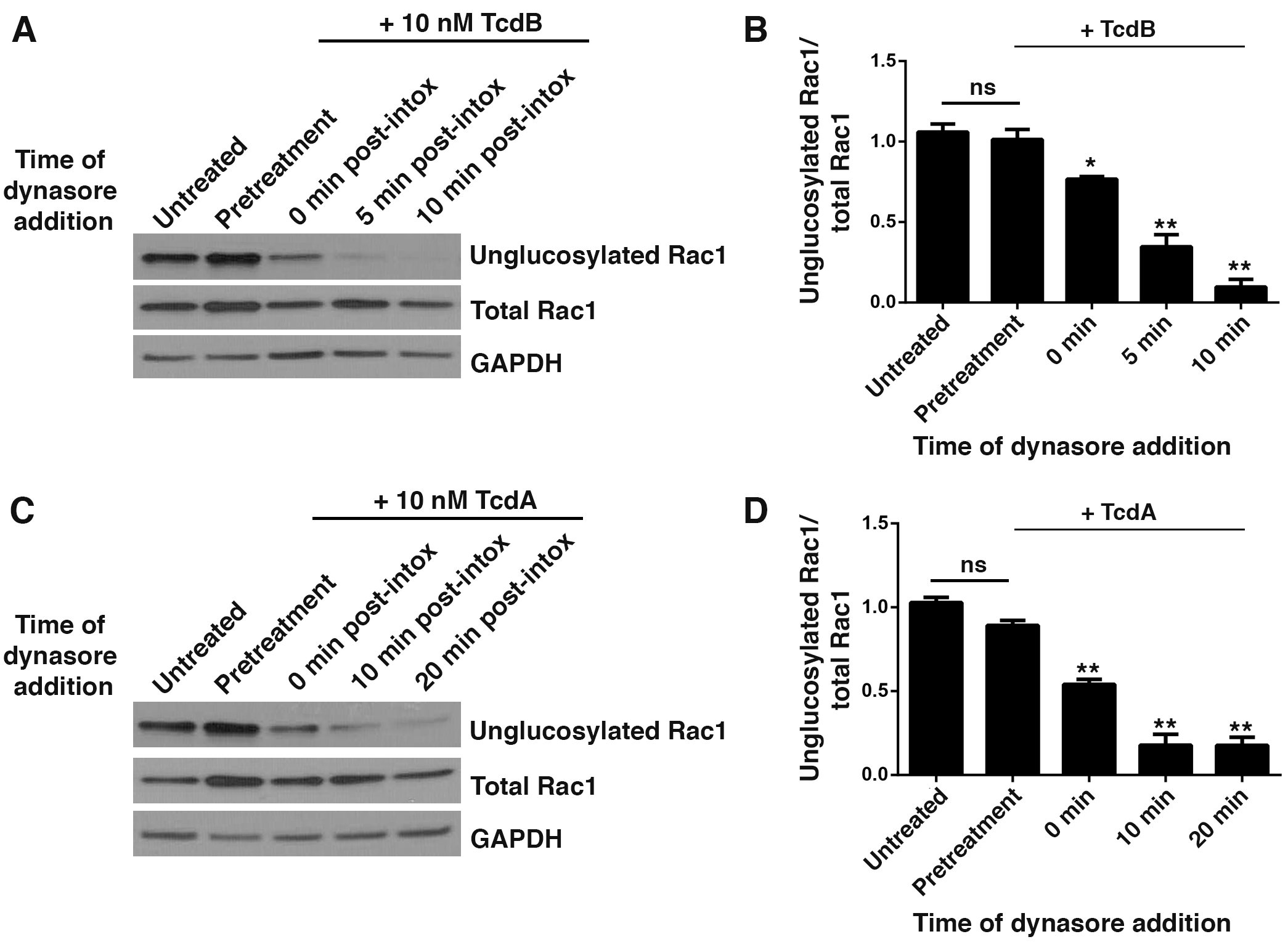

Supplement: S5 Fig — Rac1 glucosylation assays were performed with 10 nM TcdB (A) or TcdA (C) as described in Fig 2B but the time of addition of dynasore was varied. Dynasore was added 1 h prior to toxin treatment (pretreatment), or at the same time as toxin (0 min post-intox), or at various times post-intoxication. (B) and (D) Three replicates of the experiments shown in panels A and C were quantified by densitometry and represented as the ratio of unglucosylated and total Rac1 levels. Results reflect the mean and SEM, and were analyzed using a one-way ANOVA. p-values were generated using Dunnett’s multiple comparisons test in GraphPad Prism. *p<0.05; **p<0.005; ns, not significant. (TIF) [file ppat.1006070.s005.tif]

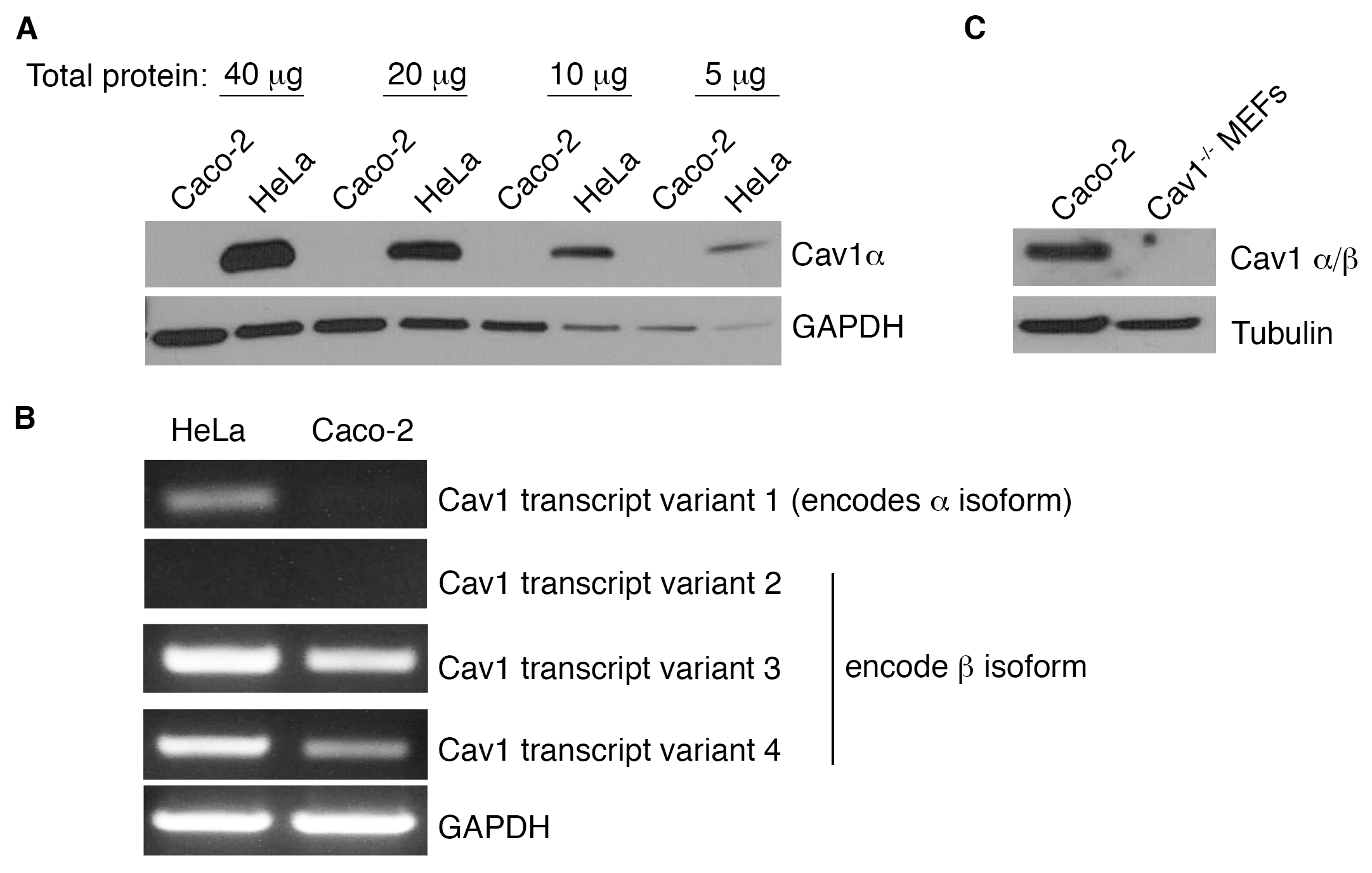

Supplement: S6 Fig — (A) Western blots of whole cell lysates from HeLa and Caco-2 cells probed with antibodies against the α isoform of caveolin-1 (sc-894) and GAPDH. (B) Total RNA from HeLa and Caco-2 cells were subjected to RT-PCR analyses to determine the mRNA expression of caveolin-1 transcript variants. GAPDH was amplified as a loading control. (C) Western blots of whole cell lysates from Caco-2 and caveolin1-/- mouse embryonic fibroblast (MEF) cells probed with antibodies against caveolin-1 (both isoforms, BD biosciences) and tubulin (loading control). (TIF) [file ppat.1006070.s006.tif]

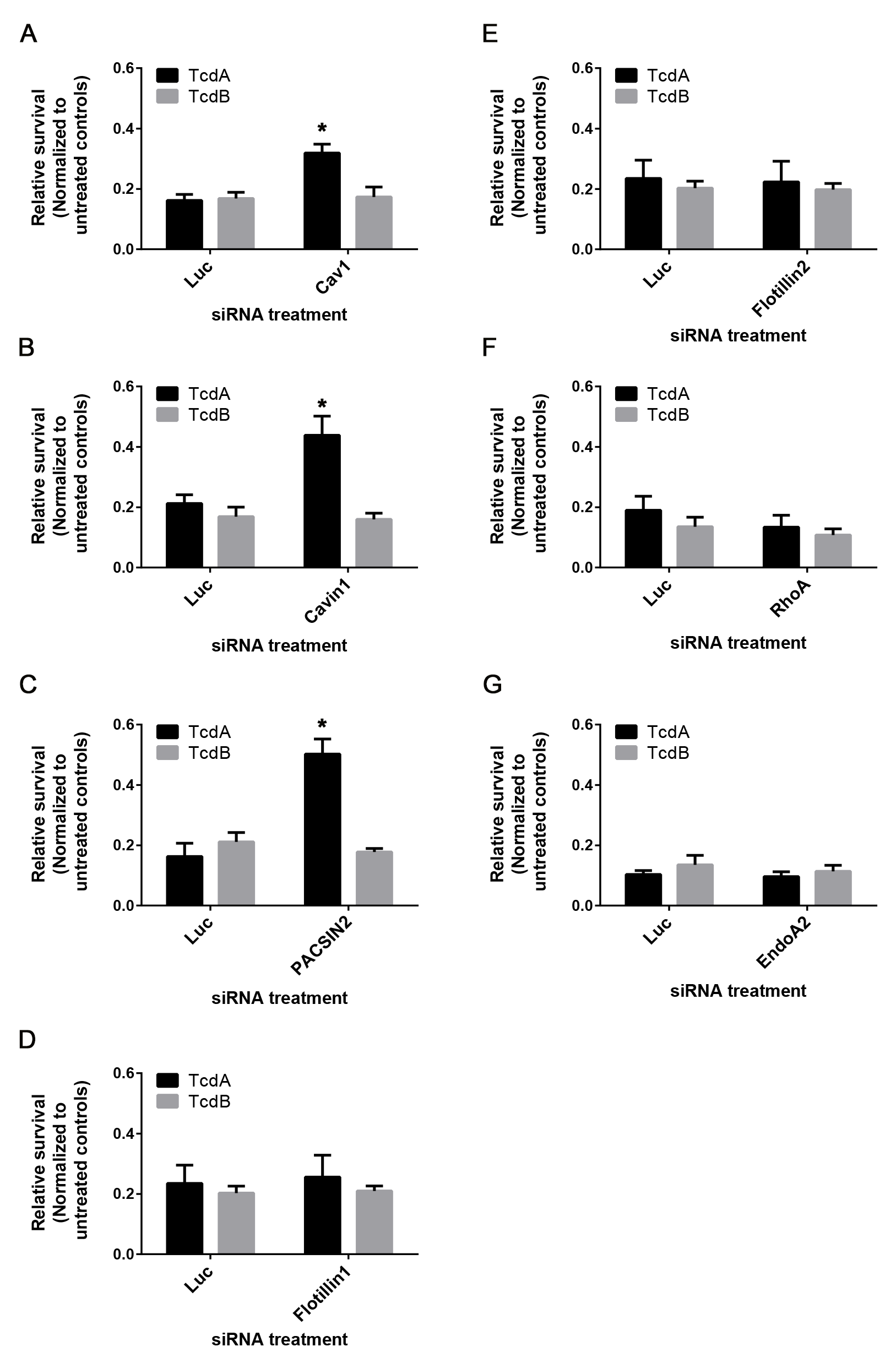

Supplement: S7 Fig — Caco-2 cells were transfected with 10 nM siRNA against Cav1 (A), Cavin1 (B), PACSIN2 (C), Flotillin1 (D), Flotillin2 (E), RhoA (F) and EndoA2 (G), exposed to 50 nM TcdA (black bars) or TcdB (gray bars) and then assayed for cellular viability using CellTiterGLO. Relative survival was obtained by normalizing the viability of treated cells to untreated (no toxin) controls. The data represent the average of at least three independent experiments performed in triplicate with the standard error of the mean indicated as error bars. Data were analyzed using t test. *p<0.05. (TIF) [file ppat.1006070.s007.tif]

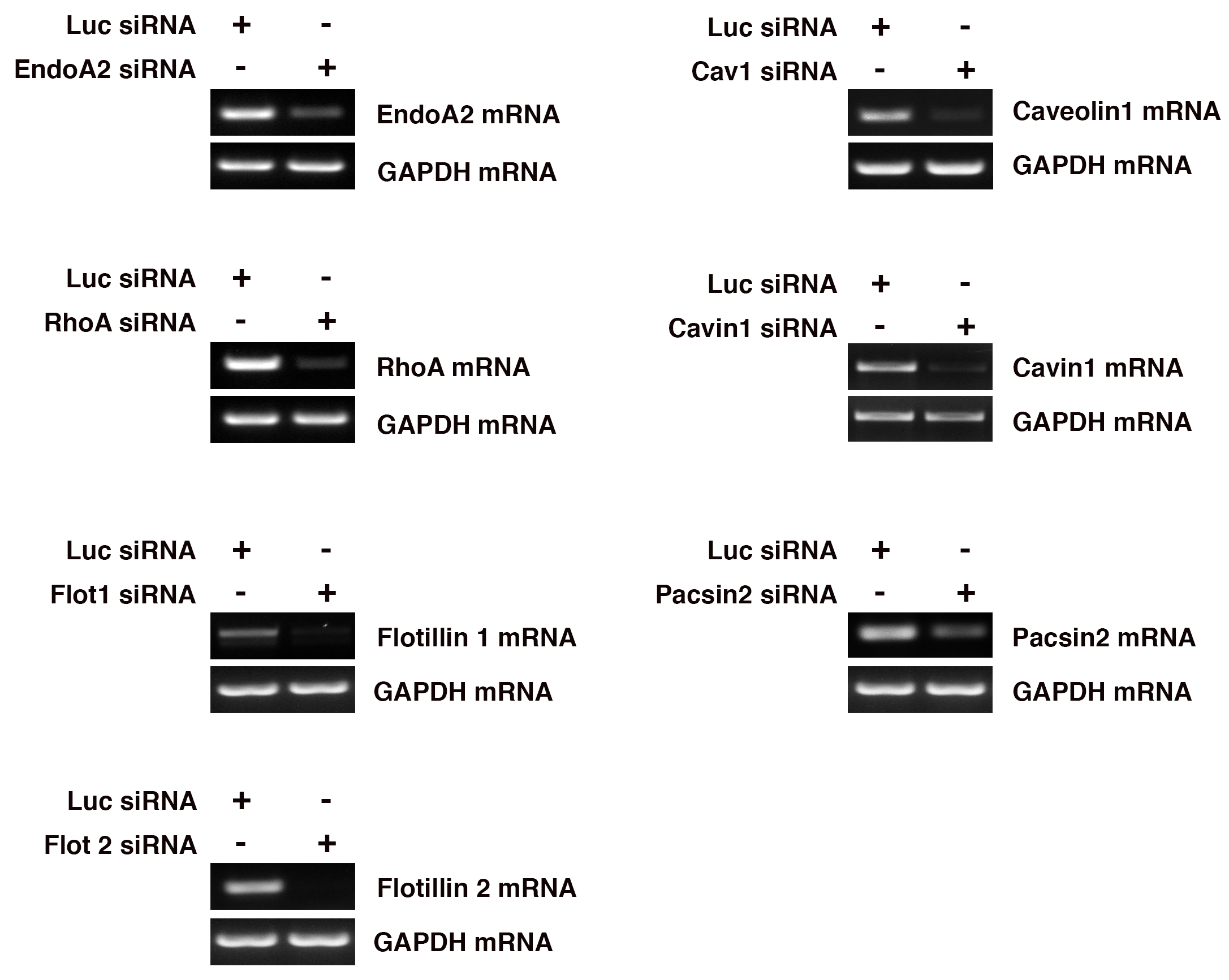

Supplement: S8 Fig — Total RNA from Caco-2 cells transfected with luciferase siRNA (Luc; non-targeting control) and siRNAs targeting various endocytic factors were subjected to RT-PCR analyses. GAPDH was amplified as a loading control. RT-PCR confirms that siRNA treatment resulted in a decrease in target mRNA expression. (TIF) [file ppat.1006070.s008.tif]

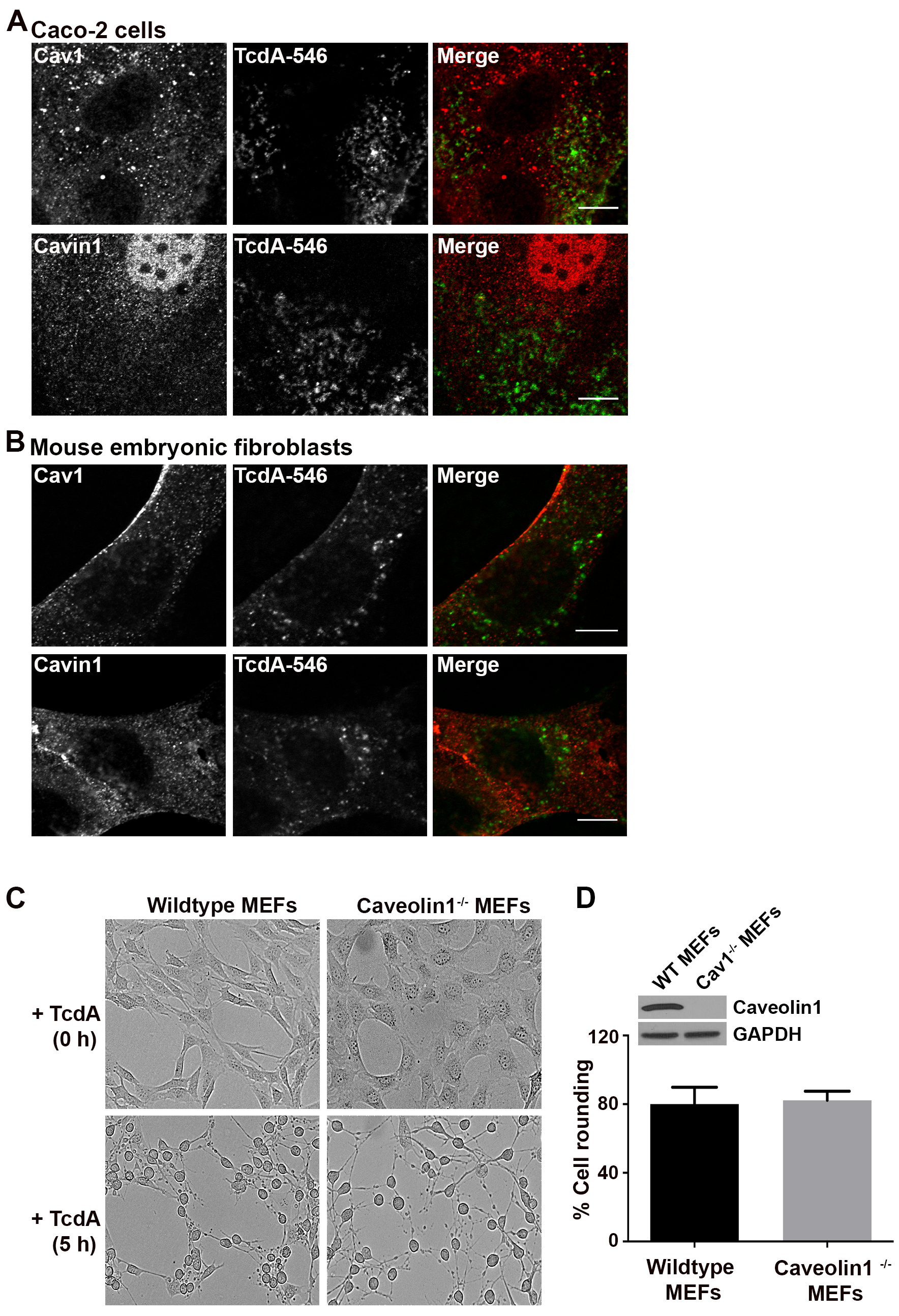

Supplement: S9 Fig — (A) TcdA does not colocalize with caveolin1 (cav1) or cavin1 in Caco-2 cells. Colocalization studies of TcdA and caveolar endocytic proteins, caveolin1 and cavin1, were performed by binding TcdA-546 to Caco-2 cells at 10°C for 45 min and shifting cells to 37°C to allow toxin uptake. After 10 min, cells were fixed and stained for caveolin1 or cavin1 and imaged using a confocal microscope. Merged images show caveolin1 or cavin1 in red and toxin in green. Scale bars, 10 μm. (B) TcdA does not colocalize with caveolin1 or cavin1 in wildtype mouse embryonic fibroblast (MEF) cells. Colocalization studies were performed in wildtype MEFs as described in (A). Toxin internalization occurred at 37°C for 3 min. Scale bars, 10 μm. (C) Caveolin1 -/- MEFs are sensitive to TcdA-induced cell rounding. Wildtype and caveolin1-/- MEFs were challenged with 10 nM TcdA and toxin-induced cell rounding effects were monitored using an imaging-based kinetic assay as described in Materials and Methods. Representative images of cells 0 h and 5 h post-toxin treatment for each cell type are shown. (D) The percentage of rounded cells 5 h post-toxin treatment was quantified for each cell type. Data represent mean and SD of at least 900 cells from three independent experiments. Knockout of cav1 was confirmed by probing western blot of whole cell lysates from wildtype and caveolin1-/- MEFs with antibodies against cav1 and GAPDH (loading control) as shown in inset. (TIF) [file ppat.1006070.s009.tif]

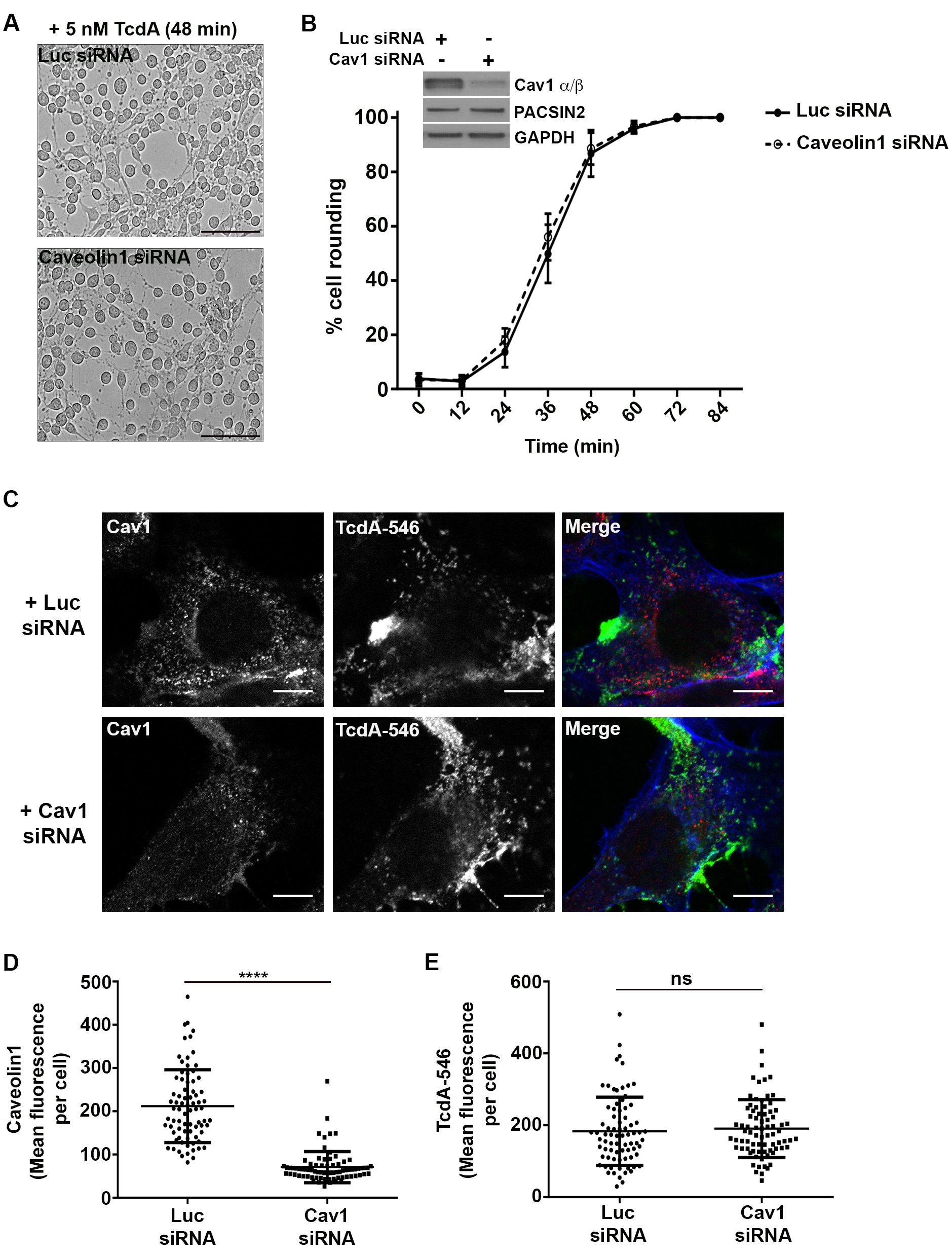

Supplement: S10 Fig — (A) and (B) Depletion of Cav1 does not affect TcdA-induced cell rounding in wildtype MEF cells. MEF cells transfected with luciferase (non-targeting) or Cav1 siRNA were challenged with 5 nM TcdA, and toxin-induced cell rounding effects were monitored using an imaging-based kinetic assay as described in Materials and Methods. Representative images of cells 48 min post-toxin treatment are shown in (A). Scale bars, 100 μm. (B) The percentage of rounded cells in each siRNA condition was quantified for the indicated time points. Data represent mean and SD of at least 1200 cells from three independent experiments. Western blots of whole cell lysates shown in inset confirms that Cav1 siRNA transfection resulted in a significant decrease in Cav1 protein levels (87.9 ± 1.2%) in cells. (C), (D) and (E). Cav1 depletion does not affect TcdA uptake in MEF cells. Wildtype MEF cells expressing luciferase (luc) or Cav1 siRNA were incubated with 50 nM TcdA-546 at 10°C for 45 min. Cells were allowed to warm up to 37°C for 2 min and then washed to remove unbound toxins and incubated with fresh media prewarmed to 37°C. Bound toxins were allowed to internalize for 9 min at 37°C. Cells were then fixed, stained for Cav1 and actin, and imaged by confocal microscopy. The images shown in (C) are representative of multiple fields imaged from two independent experiments. Merged images show Cav1 in red, TcdA-546 in green and actin (Phalloidin-647) in blue. Scale bars, 10 μm. (D) Comparison of mean fluorescence intensities of Cav1 between luc and Cav1 siRNA transfected cells. Data represent mean and SD of 77 individual cells. Student’s t test ***p<0.0001. (E) Comparison of mean fluorescence intensities of TcdA-546 between luc and Cav1 siRNA transfected cells. Data represent mean and SD of 77 individual cells and were analyzed by student’s t test. ns, not significant. Cells were chosen at random for intensity analyses. (TIF) [file ppat.1006070.s010.tif]

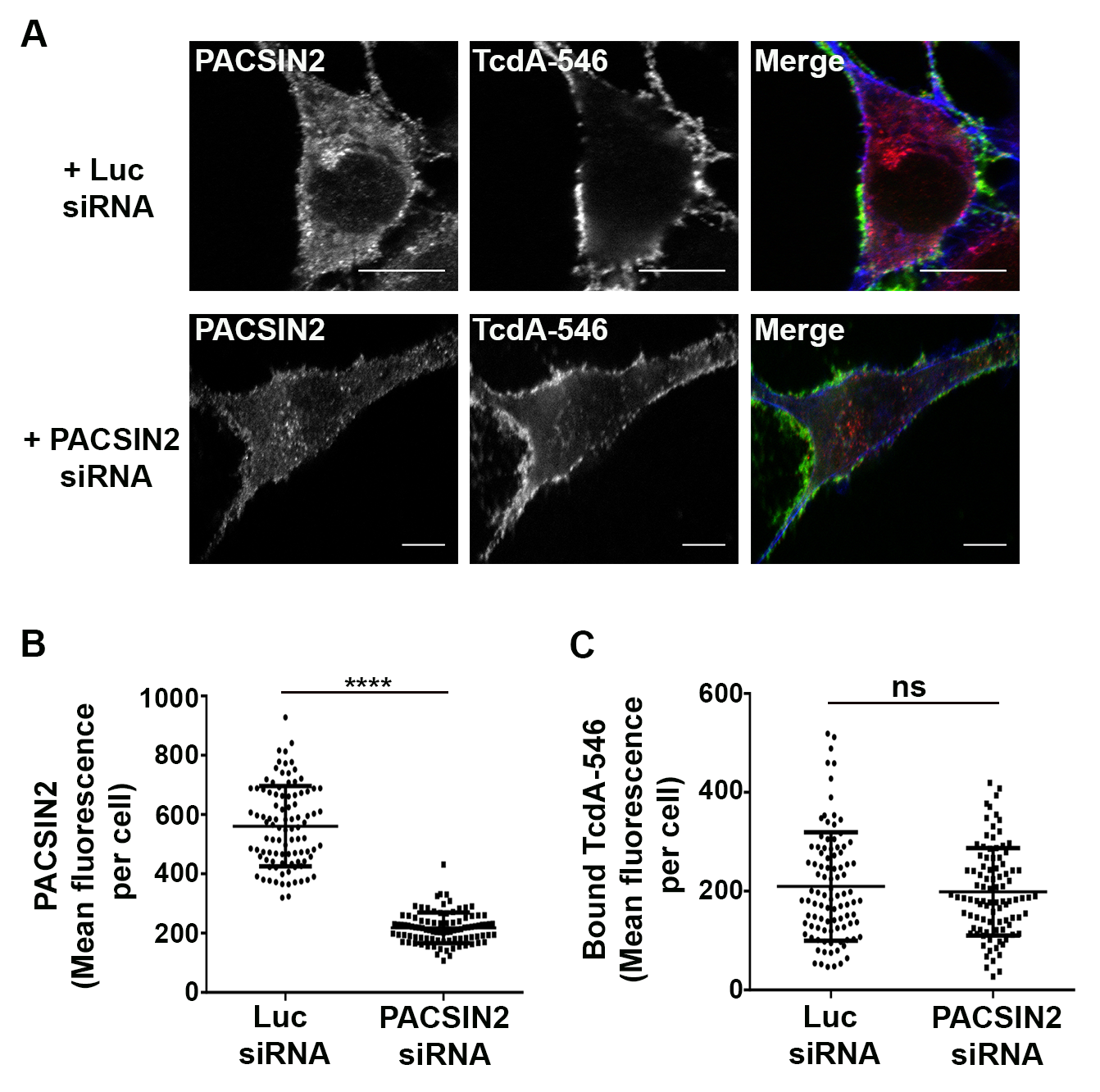

Supplement: S11 Fig — (A) Wildtype MEF cells expressing luciferase (luc) or PACSIN2 siRNA were incubated with 50 nM TcdA-546 at 10°C for 45 min. Cells were allowed to warm up to 37°C for 2 min and then washed to remove unbound toxin and fixed. Cells were stained for PACSIN2 and actin (phalloidin-647). The images shown are representative of multiple fields imaged from three independent experiments. Merged images show PACSIN2 in red, TcdA-546 in green, and actin (Phalloidin-647) in blue. Scale bars, 10 μm. (B) Comparison of mean fluorescence intensities of PACSIN2 between luc and PACSIN2 siRNA transfected cells. Data represent mean and SD of 95 individual cells. Student’s t test ***p<0.0001. (C) Comparison of mean fluorescence intensities of TcdA-546 between luc and PACSIN2 siRNA transfected cells. Data represent mean and SD of 95 individual cells and were analyzed by student’s t test. ns, not significant. Cells were chosen at random for intensity analyses. (TIF) [file ppat.1006070.s011.tif]

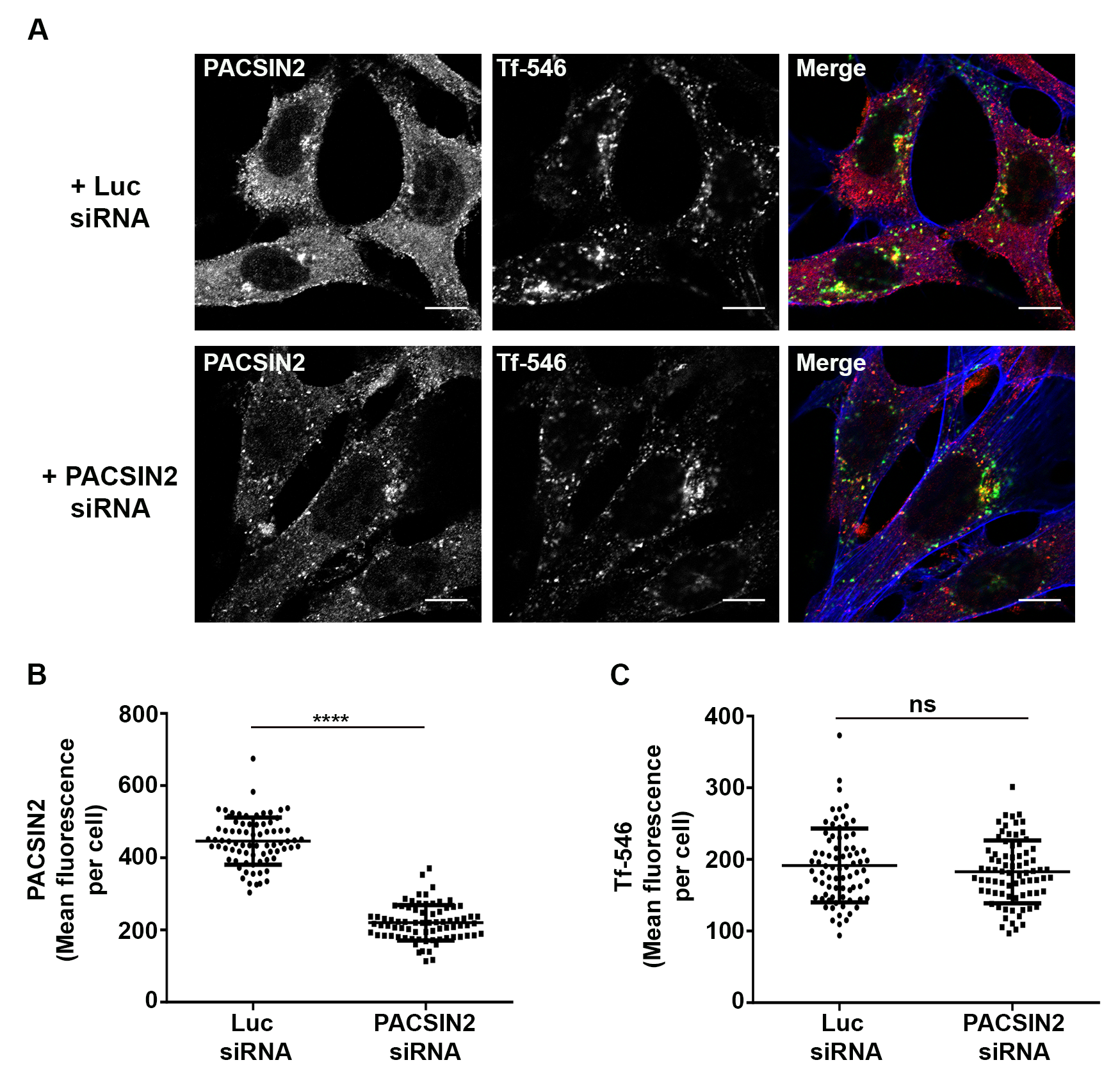

Supplement: S12 Fig — (A) Wildtype MEF cells expressing luciferase (luc) or PACSIN2 siRNA were incubated with 25 μg/ml of transferrin-alexa546 at 10°C for 45 min. Cells were switched to 37°C for 4 min, fixed and stained for PACSIN2 and actin (phalloidin-647). The images shown are representative of multiple fields imaged from two independent experiments. Merged images show PACSIN2 in red, transferrin-546 in green, and actin (Phalloidin-647) in blue. Scale bars, 10 μm. (B) Comparison of mean fluorescence intensities of PACSIN2 between luc and PACSIN2 siRNA transfected cells. Data represent mean and SD of 76 individual cells. Student’s t test ***p<0.0001. (C) Comparison of mean fluorescence intensities of transferrin-546 between luc and PACSIN2 siRNA transfected cells. Data represent mean and SD of 76 individual cells and were analyzed by student’s t test. ns, not significant. Cells were chosen at random for intensity analyses. (TIF) [file ppat.1006070.s012.tif]

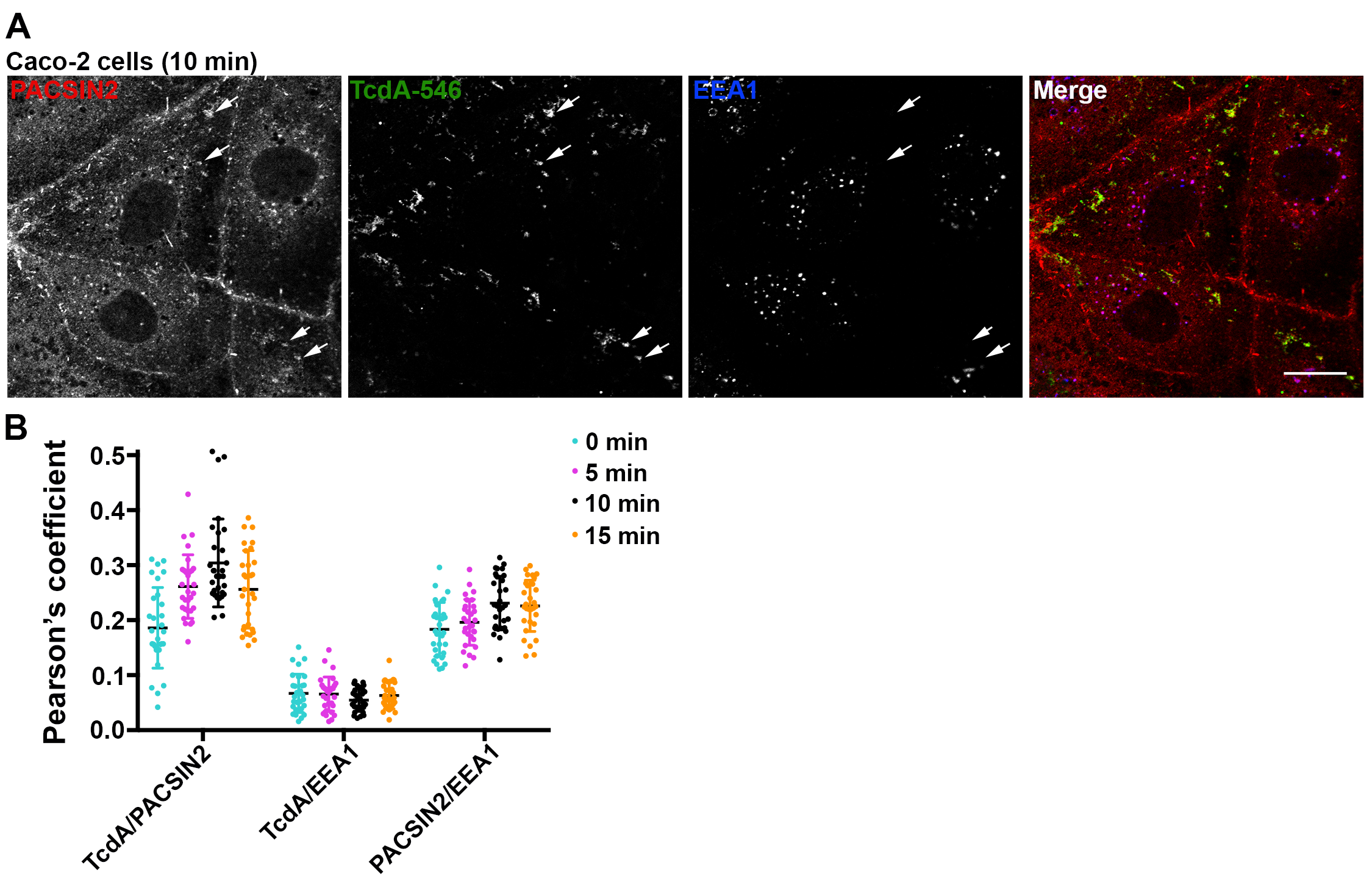

Supplement: S13 Fig — (A) Caco-2 cells on glass coverslips were allowed to bind 50 nM TcdA-546 for 45 min at 10°C. Unbound toxin was removed, and cells were shifted to 37°C to allow internalization of toxin for 0, 5, 10 or 15 min. Cells were fixed, stained for PACSIN2 and early endosomal antigen 1 (EEA1) and analyzed by confocal microscopy. Merged images show PACSIN2 in red, toxin in green and EEA1 in blue. Yellow puncta in merged images denote TcdA- and PACSIN2-positive structures. Pink punta denote PACSIN2-positive endosomes. Scale bars, 20 μm. The arrowheads in the images highlight representative regions that are positive for TcdA and PACSIN2 but not EEA1. The images shown are from the 10 min time point and are representative of multiple fields imaged from two independent experiments. (B) Pearson’s correlation coefficient to assess the extent of colocalization between PACSIN2, EEA1 and TcdA-546 at 0, 5, 10 and 15 min post-entry. Data represent mean and SD of 30 individual cells. (TIF) [file ppat.1006070.s013.tif]

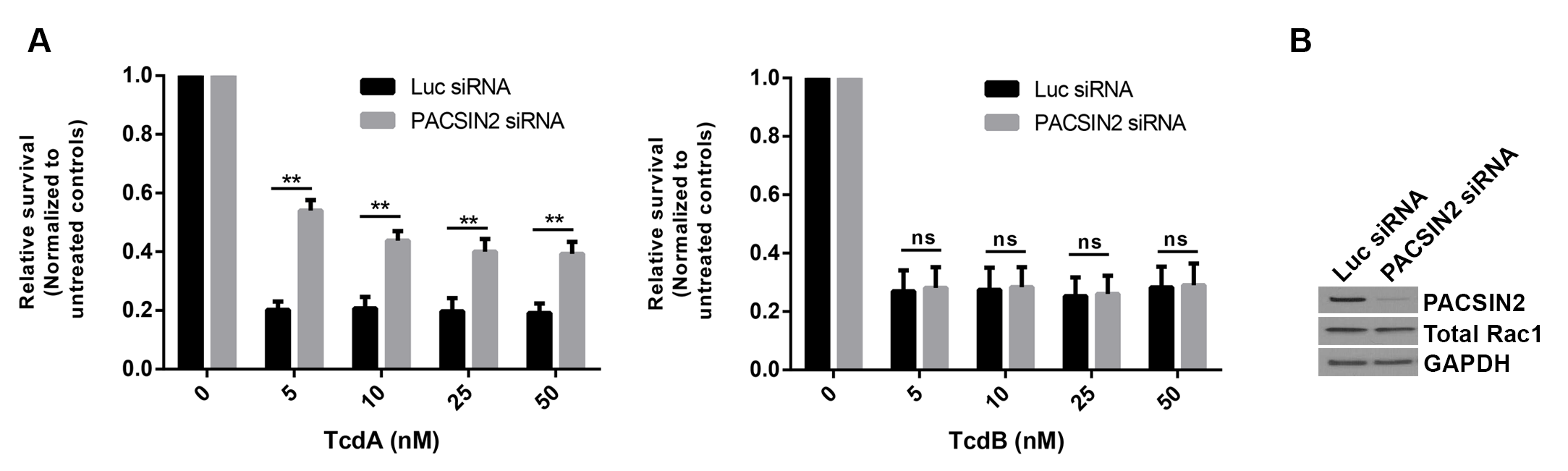

Supplement: S14 Fig — (A) Caco-2 cells were transfected with 10 nM siRNA against PACSIN2 or luciferase (Luc; non-targeting control) and then intoxicated with indicated concentrations of TcdA or TcdB. ATP levels were determined using CellTiterGlo and normalized to signal from untreated cells to assess the relative survival of cells post-toxin treatment. Results represent the mean and SEM of three independent experiments. Data were analyzed using two-way ANOVA, and p-values were generated using Sidak’s multiple comparisons test in GraphPad Prism. **p<0.005; ns, not significant. (B) Western blot of whole cell lysates from siRNA-expressing Caco-2 cells probed with antibodies against PACSIN2, total Rac1 and GAPDH (loading control). PACSIN2 siRNA resulted in 94.4 ± 4.4% reduction in PACSIN2 protein levels by densitometry. (TIF) [file ppat.1006070.s014.tif]

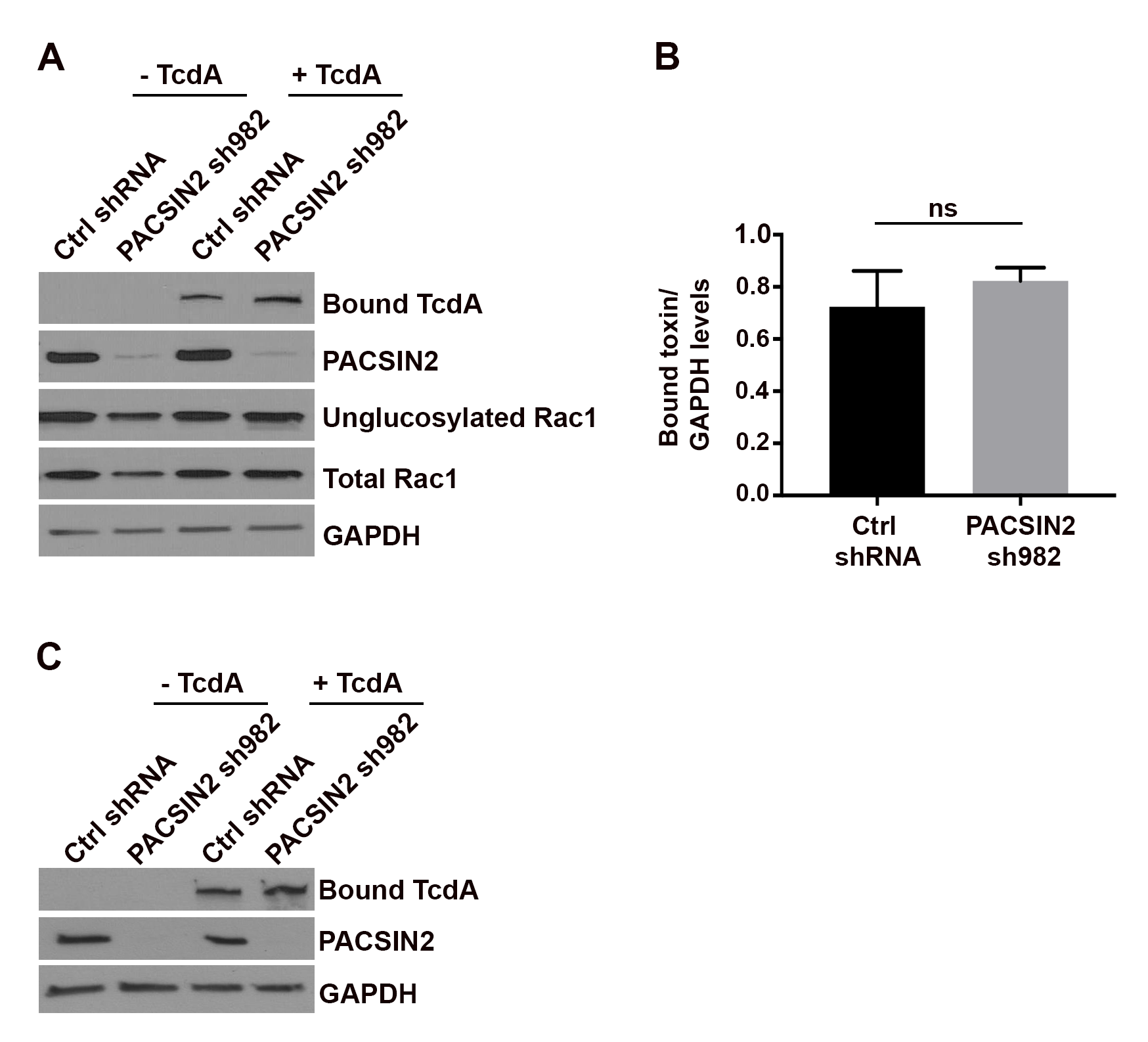

Supplement: S15 Fig — (A) Caco-2 monolayers expressing ctrl shRNA and PACSIN2 sh982 were allowed to bind 30 nM TcdA at 10°C. Whole cell lysates were prepared for SDS PAGE and Western blot. The blot was probed with antibodies against TcdA CROPs, PACSIN2, unglucosylated Rac1, total Rac1 and GAPDH. Cells that did not receive any toxin were used as a control. (B) Experiments shown in (A) were quantified by densitometry and represented as the ratio of bound toxin and GAPDH levels. Results reflect the mean and SEM of three independent experiments and were analyzed using two-tailed t-test. ns, not significant. (C) Experiment was performed as in (A) with some modifications. After toxin binding at 10°C, cells were switched to 37°C for 4 min to warm the cells to 37°C. Cells were then washed with PBS prewarmed to 37°C to remove unbound toxins and collected for lysis and western blotting. (TIF) [file ppat.1006070.s015.tif]

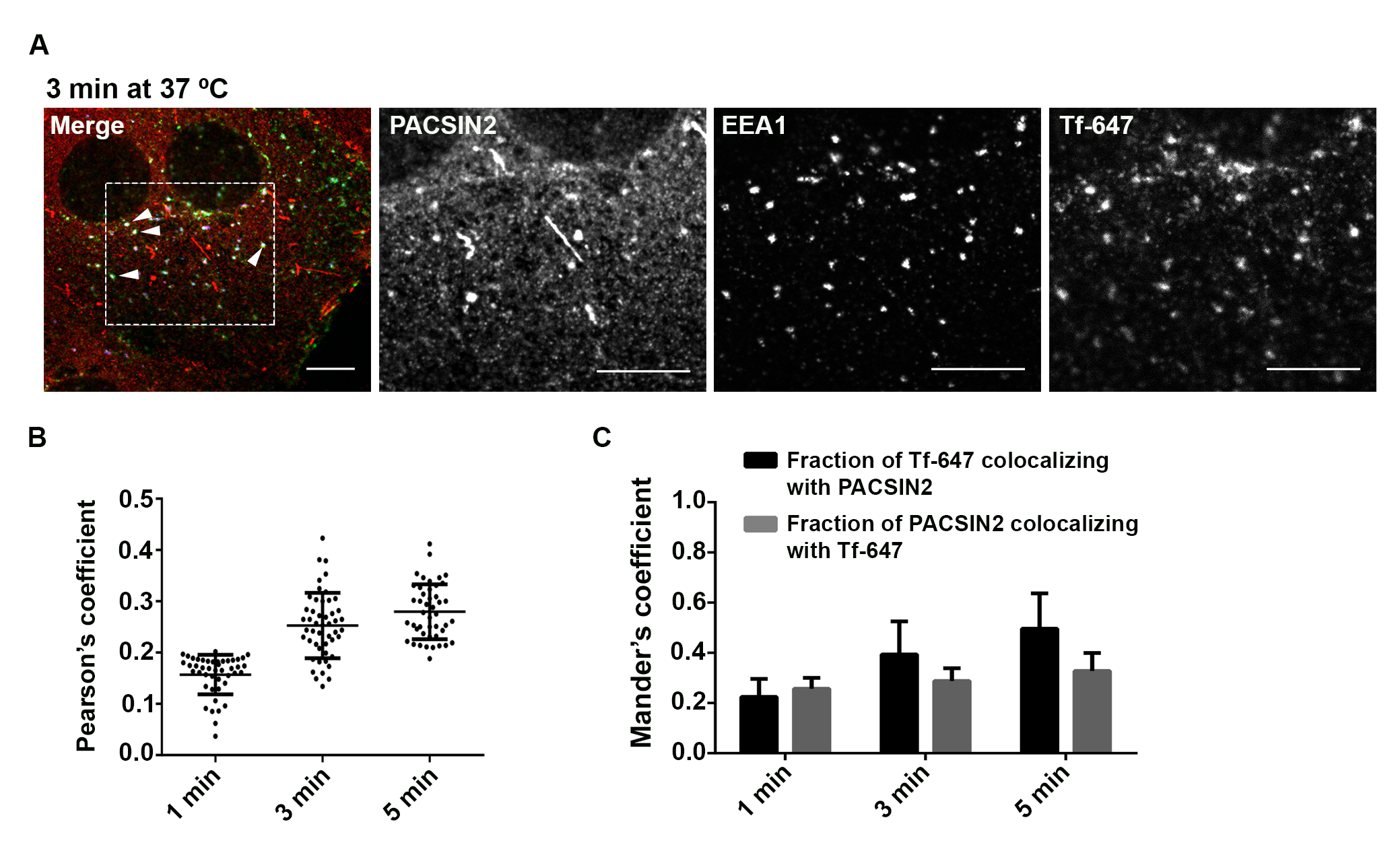

Supplement: S16 Fig — (A) Caco-2 cells on glass coverslips were allowed to bind 25 μg/ml of transferrin-alexa647 for 45 min at 10°C. Cells were shifted to 37°C to allow internalization for 1, 3 and 5 min. Cells were fixed, stained for PACSIN2 and early endosomal antigen 1 (EEA1) and analyzed by confocal microscopy. Merged images show PACSIN2 in red, transferrin in green, and EEA1 in blue. Scale bars, 10 μm. The arrowheads in the images highlight representative regions that are positive for transferrin, PACSIN2 and EEA1. The images shown are from the 3 min time point and are representative of multiple fields imaged from two independent experiments. (B) Pearson’s correlation coefficient to assess the extent of colocalization between transferrin and PACSIN2. Data represent mean and SD of at least 43 individual cells. (C) Mander’s coefficient to assess the fraction of transferrin colocalizing with PACSIN2 and vice versa. A value of 1.0 indicates 100% overlap between the two colors. Data represent mean and SD of 43 individual cells. Cells were chosen at random for colocalization analyses. (TIF) [file ppat.1006070.s016.tif]

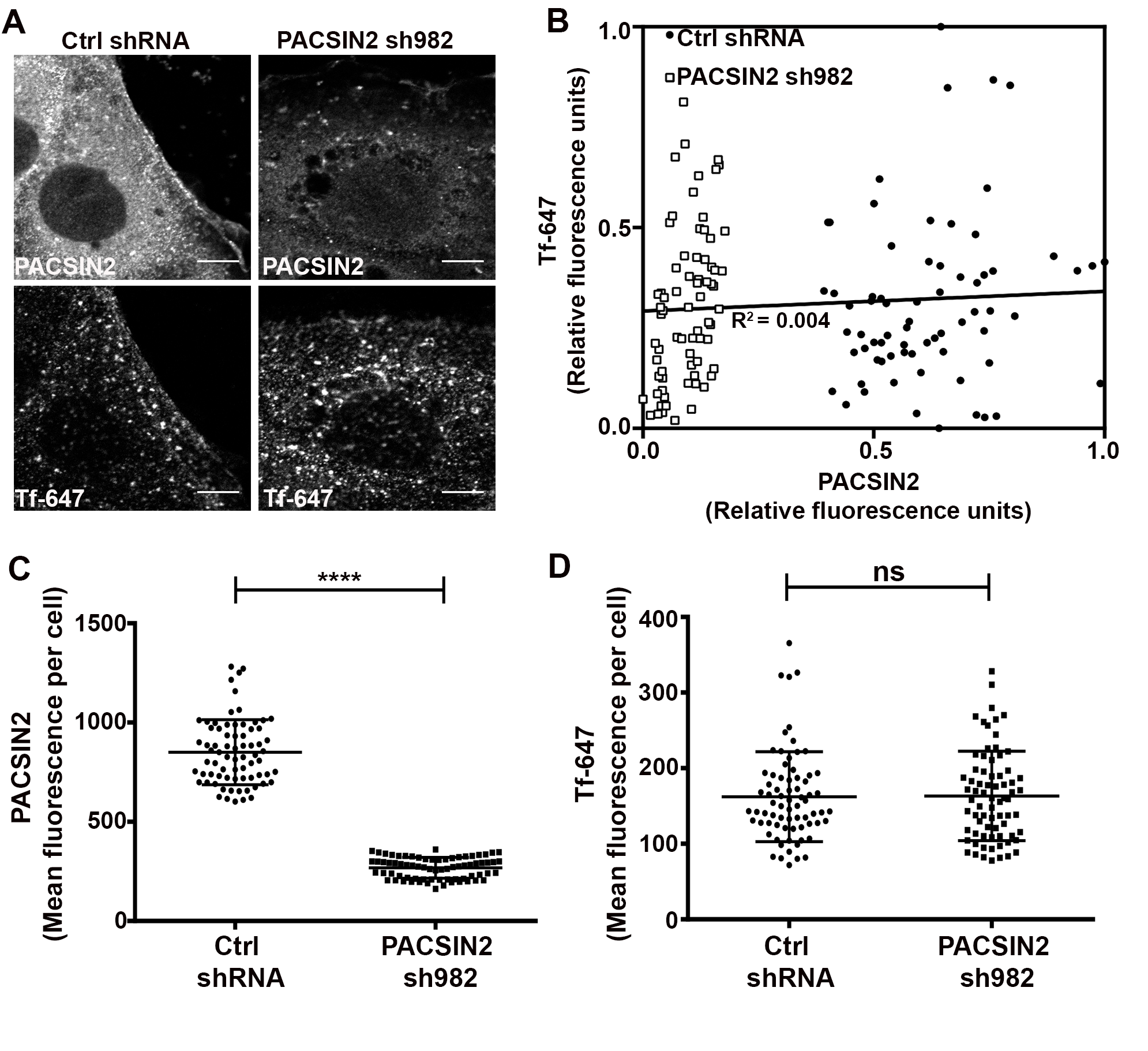

Supplement: S17 Fig — (A) Caco-2 cells expressing non-targeting shRNA (Ctrl shRNA) or shRNA 982 targeting PACSIN2 were incubated with 25 μg/ml transferrin-647 (Tf-647) at 10°C for 45 min. Cells were shifted to 37°C to allow internalization. After 5 min, cells were washed, fixed, stained for PACSIN2 and imaged by confocal microscopy. PACSIN2 and Tf-647 staining from ctrl shRNA and sh982 expressing cells are shown. Scale bars, 10 μm. The images shown are representative of multiple fields imaged from three independent experiments. (B) Scatter plot of the relative fluorescence intensities of PACSIN2 and Tf-647 in ctrl shRNA (black circles) and PACSIN2 sh982 (white squares) expressing cells. Each data point represents an individual cell. A total of 72 cells per condition were chosen at random for analyses. Linear regression analysis was performed in GraphPad Prism and indicates a lack of correlation between PACSIN2 and Tf-647 levels in cells. (C) Comparison of mean fluorescence intensities of PACSIN2 between ctrl shRNA and PACSIN2 sh982 expressing cells. Data represent mean and SD of 72 individual cells. Student’s t test ***p<0.0001. (D) Comparison of mean fluorescence intensities of Tf-647 between ctrl shRNA and PACSIN2 sh982 expressing cells. Data represent mean and SD of 72 individual cells and were analyzed by student’s t test. ns, not significant. (TIF) [file ppat.1006070.s017.tif]

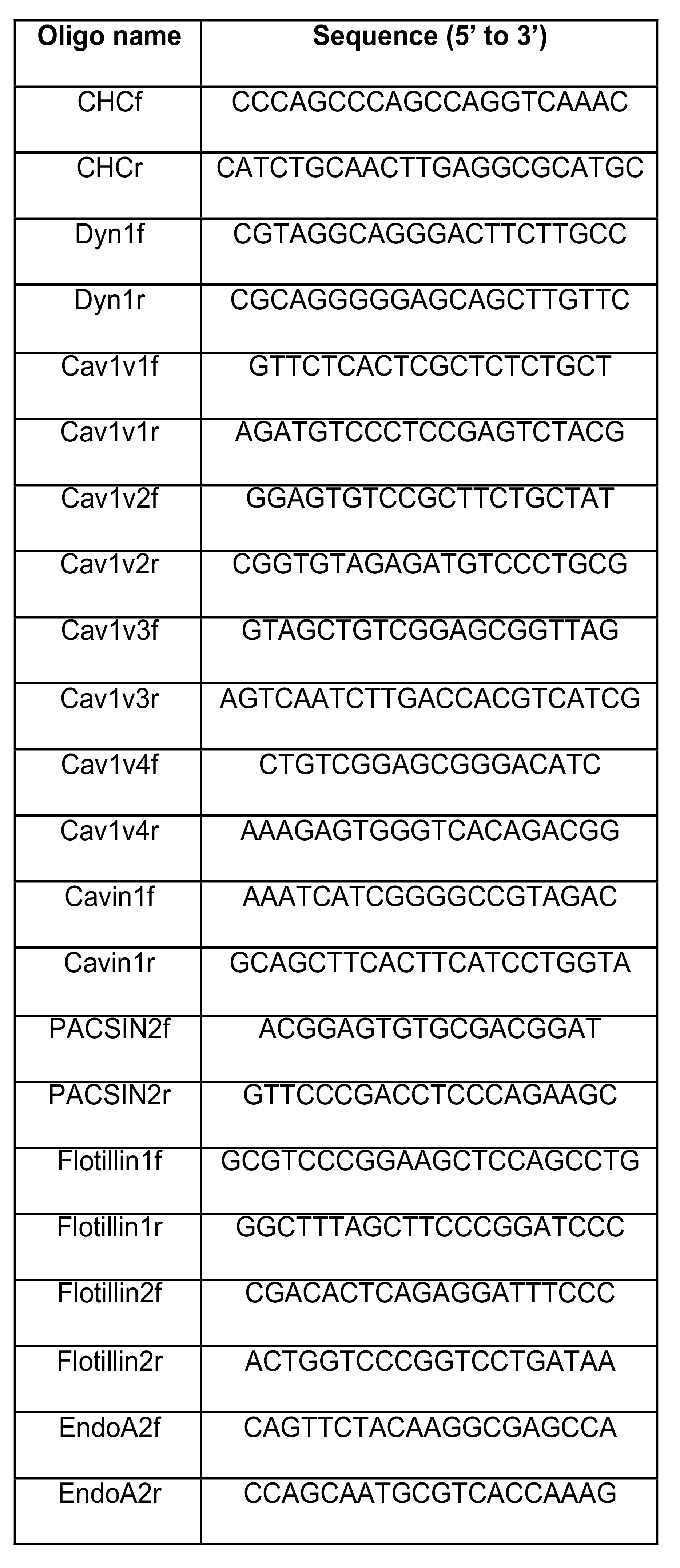

Supplement: S1 Table — (TIF) [file ppat.1006070.s018.tif]
